# Diversification in the HIV-1 Envelope Hyper-variable Domains V2, V4, and V5 and Higher Probability of Transmitted/Founder Envelope Glycosylation Favor the Development of Heterologous Neutralization Breadth

**DOI:** 10.1371/journal.ppat.1005989

**Published:** 2016-11-16

**Authors:** S. Abigail Smith, Samantha L. Burton, William Kilembe, Shabir Lakhi, Etienne Karita, Matt Price, Susan Allen, Eric Hunter, Cynthia A. Derdeyn

**Affiliations:** 1 Yerkes National Primate Research Center, Emory University Atlanta, Georgia, United States of America; 2 Emory Vaccine Center, Emory University Atlanta, Georgia, United States of America; 3 Zambia Emory HIV Research Project, Lusaka, Ndola and Kitwe, Zambia; 4 Projet San Francisco, Kigali, Rwanda; 5 International AIDS Vaccine Initiative (IAVI), New York, New York, United States of America; 6 UCSF Department of Epidemiology and Biostatistics, San Francisco, California, United States of America; 7 Department of Pathology and Laboratory Medicine, Emory University Atlanta, Georgia, United States of America; University of Zurich, SWITZERLAND

## Abstract

A recent study of plasma neutralization breadth in HIV-1 infected individuals at nine International AIDS Vaccine Initiative (IAVI) sites reported that viral load, HLA-A*03 genotype, and subtype C infection were strongly associated with the development of neutralization breadth. Here, we refine the findings of that study by analyzing the impact of the transmitted/founder (T/F) envelope (Env), early Env diversification, and autologous neutralization on the development of plasma neutralization breadth in 21 participants identified during recent infection at two of those sites: Kigali, Rwanda (n = 9) and Lusaka, Zambia (n = 12). Single-genome analysis of full-length T/F Env sequences revealed that all 21 individuals were infected with a highly homogeneous population of viral variants, which were categorized as subtype C (n = 12), A1 (n = 7), or recombinant AC (n = 2). An extensive amino acid sequence-based analysis of variable loop lengths and glycosylation patterns in the T/F Envs revealed that a lower ratio of NXS to NXT-encoded glycan motifs correlated with neutralization breadth. Further analysis comparing amino acid sequence changes, insertions/deletions, and glycan motif alterations between the T/F Env and autologous early Env variants revealed that extensive diversification focused in the V2, V4, and V5 regions of gp120, accompanied by contemporaneous viral escape, significantly favored the development of breadth. These results suggest that more efficient glycosylation of subtype A and C T/F Envs through fewer NXS-encoded glycan sites is more likely to elicit antibodies that can transition from autologous to heterologous neutralizing activity following exposure to gp120 diversification. This initiates an Env-antibody co-evolution cycle that increases neutralization breadth, and is further augmented over time by additional viral and host factors. These findings suggest that understanding how variation in the efficiency of site-specific glycosylation influences neutralizing antibody elicitation and targeting could advance the design of immunogens aimed at inducing antibodies that can transition from autologous to heterologous neutralizing activity.

## Author Summary

HIV-1 has proven difficult to vaccinate against due to its ability to generate high levels of genetic diversity, particularly in the envelope glycoproteins. An ideal prophylactic vaccine would therefore elicit immune responses capable of blocking the full range of HIV-1 variants to which a population might be exposed. An essential component of protective immunity against HIV-1 is likely to be an antibody response that is capable of neutralizing genetically diverse HIV-1 viral variants. In an effort to understand how this type of ‘broad’ antibody response develops during natural HIV-1 infection, a large cohort study recently found that certain factors, such as high viral load, HLA-A*03 genotype, and subtype C infection, were correlated with the development of greater neutralization breadth. Here we investigated the viral envelope proteins and antibody responses in early infection in a small subset of individuals from two of the African sites included in the larger cohort study. We found that a marker for the efficiency of envelope glycosylation in the infecting viral variant was strongly correlated with the development of antibodies with greater neutralization breadth. We also found that extensive viral changes in the V2, V4, and V5 regions of the envelope gp120 protein, were strongly associated with the development of antibodies with greater neutralization breadth. Based on these results, we propose that more efficient glycosylation of the envelope protein of the infecting viral variant elicits neutralizing antibodies that drive early and complex amino acid changes in gp120, which triggers the development of antibodies whose neutralization breadth can be augmented over time by additional viral and host factors. These findings suggest that a better understanding of the efficiency of envelope glycosylation in HIV-1 could inform current vaccine strategies aimed at eliciting antibodies with neutralization breadth.

## Introduction

HIV-1 has proven difficult to vaccinate against due, in part, to its ability to generate high levels of genetic diversity. During error-prone reverse transcription, up to 3.4 x 10^−5^ mutations per base pair can be introduced, and recombination can occur between the two viral genomes contained within a single virion [1,2]. Since HIV-1 group M emerged in the human population, these diversification mechanisms have led to the evolution of nine genetically distinct subtypes, as well as numerous circulating recombinant forms (CRFs) [3,4]. An ideal prophylactic vaccine would therefore elicit immune responses capable of blocking the full range of HIV-1 variants to which a population might be exposed, which differs based on geography.

The HIV-1 envelope (Env) glycoprotein subunits gp120 and gp41 are the targets of neutralizing antibodies, which are perhaps the most important correlate of vaccine-mediated protection against other viral diseases. HIV-1 Env gp120 exhibits the highest amount of amino acid variation throughout the entire proteome, creating a formidable obstacle for generating neutralizing antibody-based protection [5–7]. This is exemplified by the fact that most HIV-1 infected individuals develop robust neutralizing antibodies within a few months against the infecting virus, but these antibodies are strain-specific, and lead to viral escape [8–17]. However, in a substantial fraction of infected individuals, these strain-specific antibodies evolve to acquire neutralization activity against heterologous HIV-1 Env variants over time [18–22]. To gain insight into this process, a previous study evaluated whether early Env diversity impacted neutralization breadth, which was measured at approximately 5 years post-infection in 26 HIV-1 individuals infected predominantly with subtype A [23]. In that study, diversity was measured in the Env gp120 V1-V5 region using proviral DNA-derived sequences sampled between 17 to 299 days after infection. Early Env diversity was positively associated with later development of neutralization breadth, leading the authors to propose a model in which the process of neutralization breadth begins in early infection. Indeed, others have shown that the T/F Env and/or early escape variants can trigger the development of a specific broadly neutralizing antibody lineage [15,24–26]. However, our inability to reproduce this phenomenon via vaccination reflects a significant gap in our understanding of this process.

The development of neutralization breadth during HIV-1 infection is best understood as being a complex and continuous interplay between the host immune response and the evolving viral quasispecies [15,17,24–32]. Viral replication capacity, Env diversity, super-infection, and high viral load may all contribute to the antigenic stimulation necessary to augment heterologous neutralization breadth [21,23,27,31,33–36]. Certain HIV-1 Env characteristics, often related to subtype or glycans, may also favor the development of neutralization breadth [9,19,33,36–38]. Host immunologic factors such as T follicular helper cell activity and activation within the B and T cell compartments could also modulate the development and maintenance of neutralization breadth [39–41]. MHC Class I alleles that target CD8 T cell epitopes and influence HIV-1 disease progression could also impact the development of neutralization breadth [36,42]. On the other hand, despite strong evidence that HIV-1 pathogenesis and immune activation differs between males and females [43–48], a recent study carried out by the IAVI Protocol C team of investigators was among the first to report that there was no difference in the development of neutralization between males and females [36]. In that study, 439 participants from IAVI Protocol C at nine sites across Uganda, Kenya, Rwanda, Zambia, and South Africa were evaluated for heterologous neutralization potency and breadth starting at approximately 24 months after infection. These individuals were infected predominantly with HIV-1 subtypes A1, C, and D, the proportion of which varied depending on the location of the site. The strongest correlates of neutralization breadth that emerged in the IAVI cohort study were high viral load, low CD4 T cell count, subtype C HIV-1 infection, and HLA-A*03 genotype. Thus, from a broad perspective, there are viral and host factors that create a more favorable environment for neutralization breadth to develop during natural infection. However, none of these factors can fully predict whether an individual will develop strong neutralization. We therefore investigated early viral and immune events in individuals from this same cohort to determine their role in priming the development of neutralization breadth within a natural infection setting.

In the present study, we have examined features of the T/F Envs, patterns of early Env diversification, and the autologous neutralization responses in early HIV-1 infection. Our analysis revealed that T/F Envs with fewer NXS-encoded glycan motifs, which are associated with less efficient N-linked glycosylation, were correlated with the development of greater neutralization breadth. Of note, a previous study reported that the opposite association was found in early Envs from a clade B cohort, suggesting that Env glycosylation could be a strong determinant of breadth in a clade-specific manner [38]. Neutralizing antibody-driven diversification in the Env gp120 V2, V4, and V5 regions was also a strong contributor to the development of heterologous neutralization breadth. Thus, our finding that correlates of neutralization breadth are present in a panel of individuals in the first weeks to months after infection suggests that this process must be initiated and perpetuated during early infection by Env and antibody interactions, but is further amplified over time by a complex constellation of additional viral and host factors [36].

## Results

### Patient neutralizing antibody responses exhibit a wide range of breadth and potency at 3 years post infection

**Fig 1:**
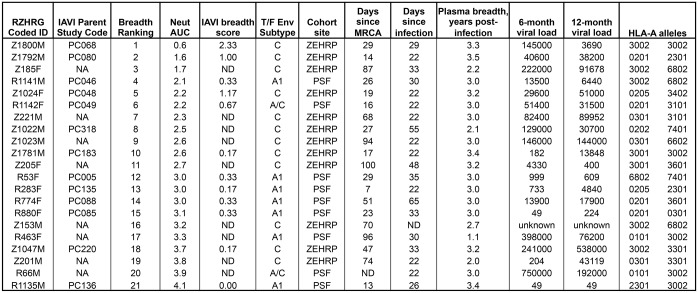
Summary of HIV-1 infected participants. The Rwanda-Zambia HIV Research Group (RZHRG) coded identification numbers for all subjects are shown, along with the associated code assigned in [36]. Individuals are listed in the order of their breadth ranking, based on the median AUC values, when plasma was tested against a panel of 12 globally representative tier 2 Envelopes (Fig 2C). The International AIDS Vaccine Initiative (IAVI) breadth score is taken from [36]. Relevant information is shown in subsequent columns, including the subtype of the T/F Env: cohort site (Zambia-Emory HIV Research Project or ZEHRP; Projet San Francisco or PSF): days since most recent common ancestor (MRCA) calculated using the Los Alamos HIV Database tool Poisson Fitter v2 [58]: days since infection estimated using methods described by [59]; time point in years post-infection when breadth was assessed; as well as the 6- and 12-month plasma viral loads (copies per ml) and HLA-A alleles determined as described in [60]. NA indicates not applicable; ND indicates not determined.

As the first step to understanding the development of neutralization breadth, we characterized the T/F Env variants, early longitudinal Env variants, and the autologous neutralizing antibody response in HIV-1 infected individuals from Lusaka, Zambia (n = 12) and Kigali, Rwanda (n = 9). We have reported that early autologous neutralizing antibody responses that develop in individuals in these cohorts are often potent but are invariably specific for the infecting strain [9–12]. Indeed, in the parent IAVI cohort study, heterologous neutralization was detected in less than 2% of individuals at 24 months post-infection [36]. The ability to neutralize heterologous Env variants that the immune response has not encountered, loosely defined as neutralization breadth, has been shown to develop in plasma by around 2–4 years after HIV-1 infection, in anywhere from 10 to 80% of individuals, depending on the nature of the cohort, timing of sample collection, and the methods used to evaluate and quantify breadth [18,21,22,33,34,36,41,49,50]. In agreement with these findings, neutralization scores indicative of breadth in the IAVI study were generally first observed at a mean of 3.5 years [36]. Therefore, to assess the level of neutralization breadth that developed in our smaller subset of individuals, we independently evaluated plasma samples collected at a median of 3.0 years (ranging from 1.1 to 3.5 years) after the first p24 antigen positive test (Fig 1; collectively referred to as 3-year plasma) for their capacity to neutralize a panel of 12 globally representative, tier 2 Env pseudoviruses, from a parent panel of 219 HIV-1 Env variants, as described by [51]. Numerous other studies have characterized HIV-1 plasma neutralization breadth using similar panels of 6 to 12 genetically diverse Env variants [11,36,40,50–56]. Since neutralization by the plasma samples at the lowest dilution tested (1:20) did not always achieve 50% inhibition, we calculated the Area Under the Curve (AUC) for each plasma-Env combination (21 plasma samples x 12 Envs) for a total of 252 infectivity curves each containing a series of 5-fold dilutions. A heatmap of the neutralization AUC values is shown in S1A Fig. Neutralization IC50 titers were also calculated for each plasma-Env combination, and are provided in a heatmap shown in S1B Fig. These parameters were highly correlated as expected (Spearman’s r = -0.99, p < 0.0001). Using this approach, plasma samples with the most potent neutralizing activity yielded the smallest AUCs, while large AUC values were observed for poor neutralizers, thus inversely related to the IC50 titer dilution. The median AUC was calculated for each individual’s plasma S1A Fig and used as a continuous variable to rank neutralization breadth [51,57].

**Fig 2:**
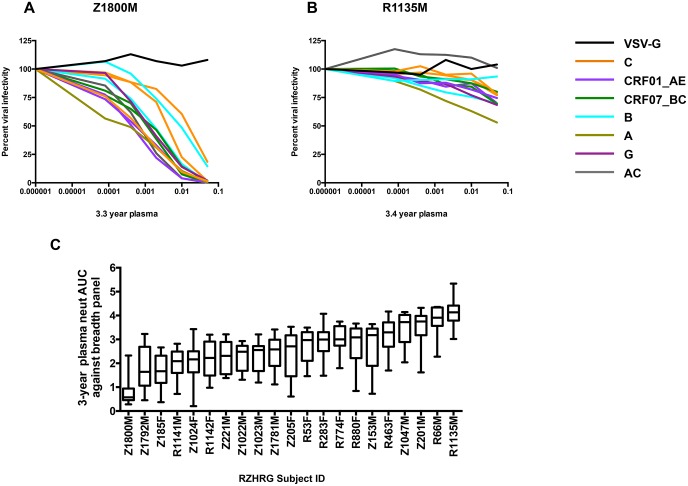
Evaluation of heterologous neutralization breadth in plasma samples from 21 recently HIV-1 infected individuals. Infectivity curves depicting neutralization activity against a panel of 12 global reference Env pseudoviruses described in [51] in the TZM-bl assay are shown for subjects Z1800M **(A)** and R1135M **(B)**. These two subjects were chosen to demonstrate the highest and lowest level of neutralization breadth. Each curve represents neutralization of one of the 12 reference Envs, and is color coded by the subtype or circulating recombinant form (CRF) designation, as indicated in the key. Neutralization of VSV-G pseudotyped virus is shown as a negative control with a black curve. The y-axis indicates percent viral infectivity relative to 100%, which is the amount of luciferase produced in pseudovirus-infected TZM-bl cells in the absence of test plasma. The x-axis indicates the reciprocal dilution of the individual’s plasma plotted on a log_10_ scale. The Area Under the Curve (AUC) was calculated from each infectivity curve to quantify neutralization potency and breadth. The median and range for the neutralization AUC values calculated for each subject’s plasma against the 12 Env reference panel are in shown in (C). High AUC values indicate weak neutralization activity, while low AUC values indicate strong neutralization activity. All AUC values are shown in S1A Fig, with the accompanying IC50 titers shown in S1B Fig.

Individual plasma neutralization curves for the subjects with the highest (Z1800M) and lowest (R1135M) overall neutralization breadth against the 12 reference Envs are shown in Fig 2A and 2B. Z1800M plasma completely neutralized ten of the reference panel Envs, which included subtypes C, B, A, G, and recombinant forms CRF01_AE, CRF07_BC, and AC, with IC50 titers in the 1:100 to 1:1,000 range (Fig 2A). The neutralizing activity against the two remaining Envs did not reach 100% but was substantial. This participant was also ranked as the best neutralizer out of 439 individuals tested in the larger IAVI study [36]. In contrast, plasma from subject R1135M had very limited neutralizing activity against all panel Envs, including the clade-matched subtype A reference Env, which did not reach 50% neutralization (Fig 2B). R1135M was ranked at #372 out of 381 participants whose follow-up extended across 24 to 48 visit months in the IAVI study, and thus exhibited poor neutralization breadth in both studies [36]. Overall, the median AUC values for the 21 plasma samples ranged from high (AUC = 0.57) to low (AUC = 4.13) neutralization breadth (Fig 2C).

For the 12 Protocol C participants that were independently analyzed in the present study and in the larger IAVI study (i.e. had an IAVI breadth score from [36], shown in Fig 1), we compared the median AUC calculated here to the maximum breadth score (sMAX) assigned in the larger study, and found that the ranking of these individuals was highly correlated as expected (S2 Fig; Spearman’s r = -0.79, p = 0.002). Median AUC values were also significantly correlated to breadth scores at Protocol C visits 36 and 42 (S2 Fig; r = -0.76, p = 0.002; r = -0.65, p = 0.006, respectively). Thus, despite using a smaller and less diverse population of HIV-1 infected subjects, a continuous variable ranking system, a different reference Env panel, and evaluation of a single time point, we detected a quantitatively similar spectrum of neutralization breadth as that reported in the IAVI study.

### A lower ratio of NXS to NXT-encoded glycan motifs in the T/F Env favor the development of nAb breadth

Features of HIV-1 Env have been previously associated with the level and type of neutralization breadth that develops within an individual [9,33,38]. Indeed, in the IAVI study involving multiple HIV-1 suptypes, subtype C infection was a significant correlate of higher neutralization breadth score [36]. However, depending on the segment of the viral genome used and the timing of sample collection, subtype determination may not fully reflect the Env glycoproteins, and also may not represent the Env of the T/F virus. Here, we assigned subtypes based on the full-length T/F Env sequences, which are the antigens that initiated the autologous neutralizing antibody response. We utilized plasma and/or PBMC samples collected at a median of 28 estimated days after infection (range 22 to 65 days; Fig 1) to capture the T/F Env sequences using single genome PCR amplification (SGA) [9,61–64]. We utilized the LANL Highlighter tool (https://www.hiv.lanl.gov/content/sequence/HIGHLIGHT/highlighter_top.html) and the LANL Poisson Fitter v2 tool (http://www.hiv.lanl.gov/content/sequence/POISSON_FITTER/poisson_fitter.html) to characterize the T/F Env sequences (Highlighter plots of all T/F Env sequences are shown in S3 Fig). Between 5 and 31 T/F Env sequences were analyzed for each individual, noting that fewer sequences were available for Z185F, Z221M, Z205F, Z153M, and Z201M, the subjects that were studied prior to the initiation of Protocol C. Two individuals, R53F and Z1800M, were infected with two distinguishable variants that differed systematically at one or two polymorphisms, respectively. These observations could indicate infection by two highly similar but distinct variants from the donor quasispecies, or reflect early selection pressure on Env. Since we do not have the donor Env sequences from these individuals, we cannot distinguish between the two scenarios. Regardless, only the dominant T/F polymorphism was observed at subsequent time points for both individuals.

Overall, the analysis of T/F Env sequences suggested infection by a single variant in majority of individuals (S3 Fig). This predominance of single variant infections is consistent with our previous study of subtype A and C HIV-1 infected transmission pairs in PSF and ZEHRP, where 18 out of 20 infections were initiated by a single variant [64]. The subtype of the T/F Envs was then determined using the LANL Recombinant Identification Program (RIP) (http://www.hiv.lanl.gov/content/sequence/RIP/RIP.html) [65,66] or the REGA HIV-1 Automated Subtyping Tool (http://dbpartners.stanford.edu:8080/RegaSubtyping/stanford-hiv/typingtool/) [67]. Seven of the 9 PSF participants in Kigali were infected with an HIV-1 subtype A1 Env variant, and the remaining 2 were each infected with a unique A/C recombinant Env (S4 Fig). All 12 individuals from the ZEHRP site in Lusaka were infected with a subtype C HIV-1 Env variant. This distribution of subtypes is also consistent with our previous studies in the ZEHRP and PSF cohorts [9,62,64,68] and with the larger IAVI study [36].

**Fig 3:**
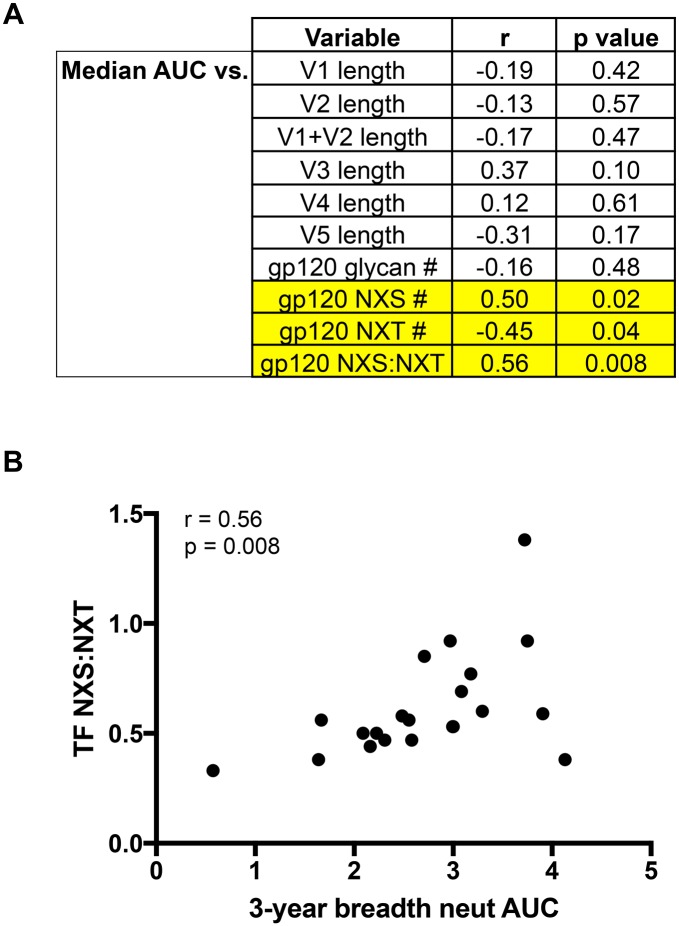
Correlation of T/F Env features with neutralization breadth. T/F Env amino acid sequence features for 21 subjects were defined using the LANL N-GlycoSite and Variable Region Characteristics online tools. A) Spearman’s correlations were performed between neutralization breadth AUC and variable loop lengths, number of total, (NXS + NXT), NXS, and NXT glycan motifs (excluding X = proline) in gp120, and the ratio of NXS motifs divided by NXT motifs. Spearman’s r values and p values are shown for each comparison. P values < 0.05 are considered significant. B) Scatter plot of median 3-year breadth AUC value vs ratio of NXS:NXT sites in the transmitted/founder Env. Spearman correlation r = 0.56, p = 0.008.

A number of studies have attempted to identify amino acid signatures, glycosylation patterns, and effects of variable loop lengths in Env that are associated with the development of neutralization breadth, as this could reveal an attractive target for vaccine immunogen design [38,69,70]. To this end, we sought to identify signatures of breadth in the T/F Envs in our cohort. Amino acid signatures of neutralization breadth previously identified by others did not emerge as correlates in our cohort [38,69]. We also examined whether the length of the variable loops or the total number of N-linked glycosylation motifs correlated with neutralization breadth (Fig 3A). Interestingly, the feature that was significantly correlated with breadth in our cohort was the ratio of NXS to NXT glycosylation sites found within the gp120 region of T/F Envs(Fig 3B, S5 Fig). N-linked glycosylation motifs are generally encoded with the amino acid motifs NXS or NXT, with X indicating any amino acid except proline; however, glycosylation at NXT occurs at a higher probability than NXS that can be as much as 40% [71,72]. Interestingly, van den Kerkhof et al previously reported a connection between the NXS/total glycans ratio in early Envs and the development of breadth in a subtype B HIV-1 infected cohort [38]. However in that study, the authors observed a higher ratio of NXS to total glycosylation sites in patients who developed breadth, postulating that a lower probability of Env gp120 glycosylation could favor breadth. We observed a contrasting effect in our mixed subtype, non-B cohort (Fig 3). While the total number of glycosylation sites in gp120 did not correlate with breadth, the ratio of NXS to NXT sites was correlated with neutralization breadth AUC (r = 0.56, p = 0.008). That is, individuals infected with T/F Env variants encoding fewer NXS glycan motifs (r = 0.50, p = 0.02) and more NXT sites (r = -0.45, p = 0.04) in gp120, and by extension a higher probability of glycosylation, were more likely to develop greater breadth. The discrepancy between van der Kerkhof et al and our study could reflect differences in the cohorts, experimental methods, Env sampling times, or time frame when neutralization breadth was measured. Furthermore, numerous biological distinctions have been reported in Env glycosylation, antigenicity, and immunogenicity, as well as in transmission and disease progression, based on HIV-1 subtype [6,9,36,46,64,68,73–80]. Indeed striking differences in conservation and variability of glycosylation sites are seen within and between HIV-1 subtypes [76,81,82]. Even the presence of relatively ‘conserved’ glycosylation positions can vary from less than 25% to over 90%, depending on subtype [76]. It is therefore not surprising that the efficiency of early Env glycosylation could impact the development of neutralization breadth in a clade-specific manner.

### Complex early Env diversification in hyper-variable regions contributes to neutralization breadth

**Fig 4:**
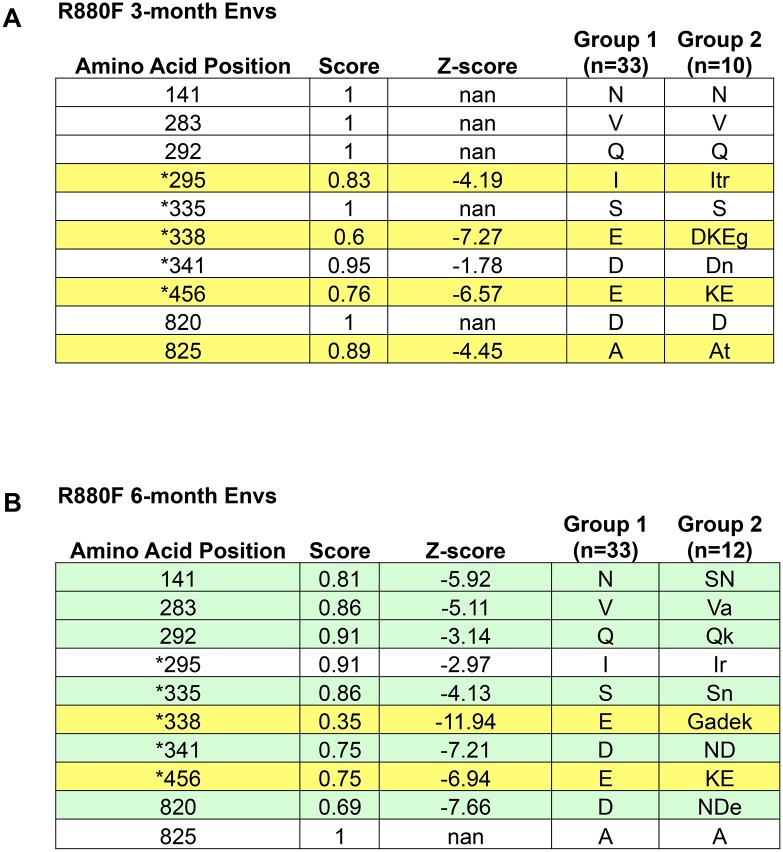
Validation of the Immunotype Diversity Index approach to quantify Env diversification using R880F. Subject R880F was chosen to validate the Sequence Harmony approach because the earliest neutralizing antibody response was previously mapped to an epitope located at the base of the V3 loop [11]. Positions indicated with an asterisk were identified and evaluated in the previous study. Thirty-three SGA derived T/F Env amino acid sequences from R880F (Group 1) were aligned with 10 sequences from 3-months post-infection **(A)**, or 12 sequences from 6-months post-infection **(B)** (Group 2). In (A), Sequence Harmony analysis identified four positions, highlighted yellow, that were significantly different (Z<-3) between the two populations at 3-months. Two of these positions were determined to be autologous neutralizing antibody escape mutations at 3-months via mutagenesis (295 and 338), whereas 456 could not be directly attributed to neutralizing antibody or cytotoxic T lymphocyte selective pressure. In (B), Sequence Harmony analysis identified two positions that remained significant from 3 months (highlighted yellow), six positions that became significant at 6 months (highlighted green), and two positions that lost significance at 6 months (not highlighted). Positions 335, 338, and 341 were all determined to be neutralizing antibody escape mutations at 6 months.

We next sought to gain insight into whether early Env diversification influenced subsequent neutralization breadth in our cohort of subjects. To understand the shifts from the T/F Env to a more diverse quasispecies due to selective pressures, we analyzed SGA derived full-length ‘early’ Env sequences derived from plasma and/or PBMC samples collected after the development of potent strain-specific neutralizing antibodies [9,11,12]. Because nucleotide-based dN/dS analyses are problematic when resampling the same population over short periods of time [83], and do not fully reflect the range of diversity that occurs within Env at the amino acid level (conservative vs. non-conservative amino acid changes, insertions and deletions, alterations to glycosylation sites), we developed and implemented a novel approach for statistically quantifying early Env diversity. First, FASTA amino acid alignments of the T/F Envs and early Envs from the same patient were subjected to Sequence Harmony analysis to identify amino acid positions that were significantly different between the two groups of sequences (Z-score ≤ -3) [38,63,84]. As a proof of concept for this approach, we first analyzed sequences from subject R880F, whose early neutralizing antibody responses and escape pathways had been previously mapped in our laboratory in great detail [11]. Sequence Harmony analysis not only confirmed our previous findings of neutralizing antibody escape mechanisms at 3-months and 6-months after infection (Fig 4A and 4B, respectively), which were characterized through extensive Env mutagenesis and neutralization assays, but also identified an additional four positions where the amino acid composition at 6-months post-infection had significantly shifted from the T/F Envs (Fig 4B). Sequence Harmony analysis was therefore performed on Env sequences from 12 subjects who had sufficient numbers of Env sequences available (minimum of 15 total sequences) from an early longitudinal plasma sample, which ranged from 3.7 to 7.7 months after infection.

Next, to take into account the biochemical nature of the identified changes in amino acid composition, we developed an Immunotype Diversity Index (IDI) that reflected the complexity of diversification that occurred in Env, particularly in highly variable regions. This approach is based on the premise that antibodies must be exposed to variation within their epitopes, i.e. immunotypes, to acquire heterologous neutralization breadth [19,27]. The IDI score was calculated as follows: For amino acid positions with Z-scores < -3, a conservative amino acid change was given 0.5 points; while a non-conservative change, an insertion or deletion, and a purifying selection event were each given 1 point. It is important to note, that this approach takes into account all SGA derived T/F Env sequences, as opposed to using a consensus T/F Env as a point of comparison. This analysis can therefore factor in the presence of amino acid diversity near the time of infection, thus allows for the identification of purifying events. Multiple amino acid changes at a single position (e.g. in R880F, position 338 shifts from E in the T/F Env to G, A, D, or K in the 6-month Env population, Fig 4B) were given appropriate points for each possible change (0.5 or 1, depending on the nature of the change), and added together. Furthermore, if the amino acid change resulted in an introduction, deletion, or shift of a glycosylation site, the significant position was given an additional point. For example, in R880F at position 335 (Fig 4A), there was a non-conservative change from S to N (1 point) that shifted a glycosylation motif (1 point), giving amino acid position 335 a total of 2 points. The final value for each identified position was multiplied by the absolute value of the calculated Z-score, to factor in the statistical significance of the change. That is, a non-conservative amino acid change that just meets the significance threshold (-3) will have 3 points, but is not weighted equally to the same change with a highly significant Z-score of -30, which will result in 30 points.

**Fig 5:**
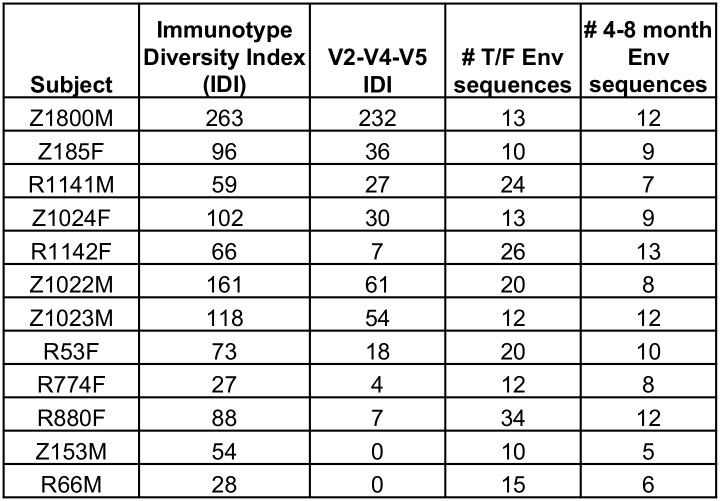
Immunotype Diversity Index scores for 12 subjects. For each position where Z-scores were less than -3 in Sequence Harmony analysis, the nature of the change, in addition to shifts in glycans and the significance of the difference, was taken into account when calculating the Immunotype Diversity Index. Changes (conservative = 0.5; non-conservative, deletions, purifying selection = 1, glycan shift or deletion = 1) were multiplied by absolute value of Z-scores to factor in significance, and added together for a final IDI. These values ranged from 28 to 263. When IDI was restricted to significant positions within V2, V4, and V5, the values ranged from 0 to 232. The number of T/F Env sequences and longitudinal Env sequences from 4–8 months included in this analysis are indicated.

**Fig 6:**
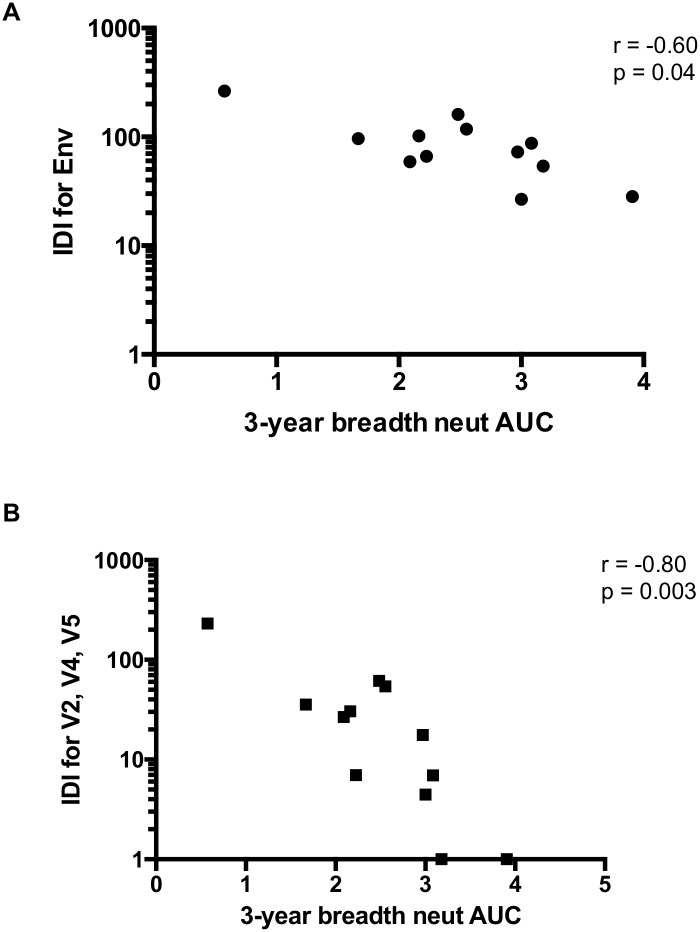
Correlation of Immunotype Diversity Index scores with neutralization breadth. **A)** Spearman’s correlation of IDI scores with median AUC breadth scores (n = 12) revealed a relationship between early Env diversity and later development of neutralization breadth (r = -0.60, p = 0.04). **B)** This correlation was stronger when IDI scores only included diversity found within the V2, V4, and V5 variable loops (r = -0.80, p = 0.003). Two patients with V2-V4-V5 scores of zero were given a value of 1 for illustrative purposes on the log10 scale.

The final IDI score was calculated for each patient by taking the sum of all points for all significant positions (Fig 5). Overall, the IDI scores varied over almost 10-fold, ranging from 263 in Z1800M to 28 in R66M, demonstrating broad variation in the amount of early Env diversity in our cohort (Fig 5). S6A Fig shows an amino acid alignment for Z1800M, with regions that factored into the IDI score highlighted, as a representative example of high Env diversity that is concentrated in V2, V4, and V5. The same is shown for R66M in S6B Fig to represent low Env diversity. When the IDI scores for full-length T/F Env were plotted against median AUC neutralization breadth scores, there was a significant negative correlation (Spearman’s r = -0.60, p = 0.04; Fig 6A). Thus, complex and dramatic amino acid changes associated with stronger selection in Env were correlated with greater breadth. However, much of the significant Env amino acid diversity was occurring in the V2, V4, and V5 regions of Env gp120 (see S6A Fig for an example). When IDI scores were calculated using only these three regions, a much stronger correlation was observed (Spearman’s r = -0.80, p = 0.003; Fig 6B). Thus, complex changes in V2, V4, and V5 of gp120 appear to play a particularly strong role in driving the development of neutralization breadth in our cohort.

### Early Env diversity is linked with selective pressure exerted by autologous neutralizing antibodies

**Fig 7:**
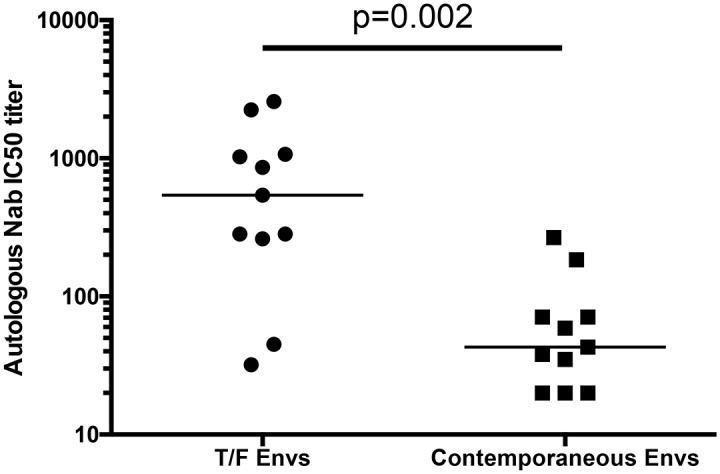
Autologous early neutralization of T/F Envs vs. contemporaneous Envs. For 11 patients, sampling allowed neutralization assays to be performed on T/F Envs, and Envs from an early time-point (range 2 to 12 months, median 5 months), with the early time-point plasma listed in Fig 8. Wilcoxon matched-pairs signed rank test revealed a significant difference between neutralization IC50 titer of the early plasma vs. T/F Envs, compared to the same plasma against the contemporaneous Envs (p = 0.002).

**Fig 8:**
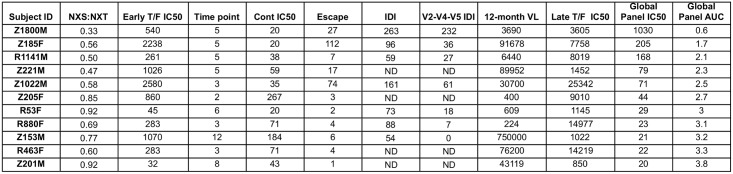
Summary of quantitative factors that potentially contribute to neutralization breadth. Autologous plasma neutralization IC50 titers for the T/F Env and contemporaneous Envs from an early time point ranging from 2 to 12 months (median 5 months) is shown for 11 patients, along with the ratio of NXS to NXT glycan sites in the T/F gp120, the magnitude of escape (T/F Env IC50 divided by contemporaneous Env IC50), full Env and V2-V4-V5 IDI values, and the 12-month plasma viral load. The neutralization IC50 titer of 3-year plasma against the autologous T/F Env and the heterologous global reference Env panel is shown, in addition to the neutralization AUC against the global reference panel.

Sequence diversity within Env during early HIV-1 infection is likely to be the result of pressure exerted predominantly by replicative capacity, cytotoxic T-cell responses, and autologous neutralizing antibodies [10–12,85–87]. For 11 of the 21 individuals, we had sufficient samples available to directly evaluate autologous neutralization against the T/F Env and contemporaneous Envs at an early time point ranging from 2 to 12 months (median of 5 months). As autologous neutralizing antibody responses are generally more potent that heterologous responses, we calculated the neutralization IC50 titers for the T/F Env and contemporaneous Envs for these 11 individuals. If we were unable to calculate the IC50 titer due to lack of neutralization activity, we used a value of 20. The autologous neutralization IC50 titers for the T/F Envs ranged from 32 to 2,580, while the titers for neutralization of the contemporaneous Envs were lower, ranging from 20 to 267 (Figs 7 and 8). As a group, the T/F Envs were significantly more sensitive to neutralization by the early autologous plasma than were the contemporaneous Envs, indicating that escape from autologous neutralizing antibodies had occurred during this time period (Fig 7, Wilcoxon matched-pairs signed rank test, p = 0.002). The magnitude of escape was then quantified by dividing the T/F IC50 titer by the contemporaneous titer. The magnitude of escape ranged from 1 (i.e. Z201M), meaning there was essentially no difference between neutralization susceptibility of the T/F Env and the contemporaneous Envs, to 112 (Z185F) (Fig 8). We also evaluated the ability of the 3-year ‘late’ plasma (that was used to determine breadth) to neutralize the T/F Env, and these IC50 titers ranged from 850 to 25,342. We next performed a non-parametric Spearman’s correlation analysis using these variables, along with the ratio of NXS:NXT sites in the T/F Env and the 12-month viral load, to examine potential relationships between these factors and the development of neutralization breadth.

**Fig 9:**
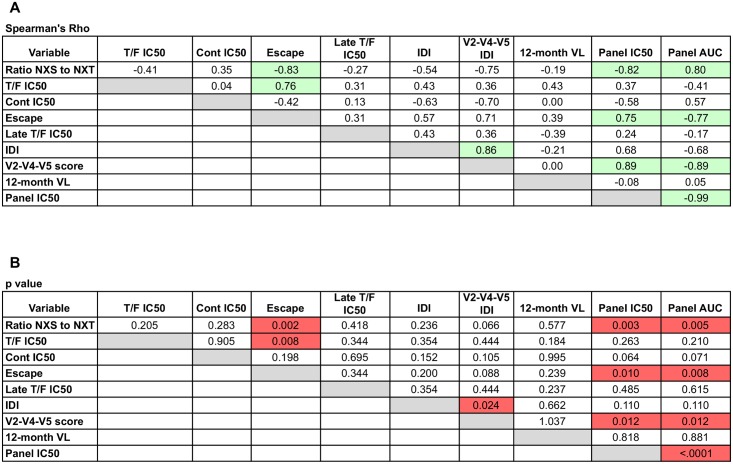
Spearman’s correlation analysis of quantitative factors and neutralization breadth. **A)** Spearman’s rho values are shown for each pairwise correlation analysis between variables. **B)** p-values are shown for each pairwise correlation analysis between variables. A p value < 0.05 was considered significant.

As expected, the neutralization IC50 and AUC values against the global reference panel were strongly inversely correlated (Fig 9; Spearman’s r = -0.99, p < 0.0001). Interestingly, even within this smaller subset of subjects, the ratio of NXS to NXT glycan motifs in the T/F Env gp120 proteins was again significantly correlated with the development of neutralization breadth using either measurement (Fig 9; Spearman’s r = -0.82, p = 0.003 for IC50; r = 0.80, p = 0.005 for AUC). The NXS:NXT ratio was also strongly and inversely correlated with the magnitude of escape (Spearman’s r = -0.83, p = 0.002). Thus, potentially higher efficiency of glycosylation in gp120 was associated with a higher magnitude of early escape from autologous neutralizing antibodies and the development of neutralization breadth. The potency of T/F Env neutralization was also correlated directly and significantly with the magnitude of escape (Spearman’s r = 0.76, p = 0.008), underscoring the importance of a dynamic relationship between the T/F Env, early neutralizing antibodies, and viral escape. The importance of this early co-evolutionary process is further substantiated by the fact that both the V2-V4-V5 IDI and the magnitude of escape were also strongly correlated with the development of greater breadth (V2-V4-V5 IDI: Spearman’s r = 0.89, p = 0.012 for IC50; r = -0.89, p = 0.012 for AUC; Escape: Spearman’s r = 0.75, p = 0.010 for IC50; r = -0.77, p = 0.008 for AUC).

## Discussion

A major goal for HIV-1 vaccine development is to induce neutralizing antibodies that are capable of broadly protecting against a globally diverse population of viral variants. To this end, various studies have attempted to identify signatures within HIV-1 Env or clinical/host factors that correlate with the development of breadth [10,21,22,31,36,38,69,77,88], on the basis that they could be targeted by immunization strategies. We postulated that factors present as early as several weeks after infection could also be determinants for subsequent development of breadth, as early Env diversity was previously linked with breadth [23]. Using a panel of individuals exhibiting a spectrum of neutralization breadth that was highly correlated with that found for the larger parent cohort [36], we analyzed the inter-dependent relationships between the T/F Env; Env diversification, neutralizing antibodies, and viral escape during the first months of infection; and development of neutralization breadth several years later. Our focus on early infection stems partly from the observation that generic correlates (i.e. high viral load, subtype C infection) cannot fully explain the development of breadth, as there are many individuals that have these characteristics but fail to develop broad heterologous neutralization capacity. In addition, understanding which early viral and immune events are associated with generating desirable antibody responses during infection could most directly inform strategies to transition from vaccine-induced strain-specific autologous neutralizing antibodies, which have been recently elicited by trimer-based immunogens [89,90], to those with broader neutralizing capacity.

Our observations suggest that the long process of developing neutralization breadth is initiated at least in part by glycosylation of the T/F Env, and is perpetuated by antibody-driven Env diversification in gp120 hyper-variable regions. Our findings are also consistent with previous reports that tracked broadening of a neutralizing antibody lineage within single individuals [24,26,27]. However, further augmentation by factors linked to viral load or disease progression may also be necessary to develop high levels of neutralization breadth, but are more difficult to translate into immunization strategies [36]. Currently, it is unknown how parameters such as viral subtype and HLA-A alleles could contribute to the broadening of the neutralizing antibody response. One possible explanation is that subtype C HIV-1 infection generates higher plasma viral loads than subtype A infection [46]. Furthermore, HLA alleles can influence the immune response to HIV-1 infection and disease progression via both innate and adaptive immune pathways in the same cohort, and thus could have durable or transient effects that act at different stages of infection [46].

Regarding the impact of the T/F Env, our finding that fewer NXS glycan motifs favor the development of neutralization breadth highlights the important and incompletely understood relationship between Env glycosylation and immunogenicity. Various models of glycan site occupancy have revealed a strong preference for glycosylation at NXT over NXS [71,72,91–94]. This could result from NXT providing a more optimal conformation of the acceptor sequence, with increased nucleophilicity of the asparagine amide group [91]. Furthermore, kinetics studies have demonstrated that the eukaryotic oligosaccharyltransferase enzyme has a higher affinity for NXT sites compared to NXS [95,96]. NXT motifs are also positively selected for in adjacent sequons, and are more likely than NXS to be glycosylated in this setting [97]. Other factors, such as the flanking sequences, tertiary protein structure, and proximity to the C-terminus of the protein, as well as the amino acid at the X position, could also influence the probability and extent of glycosylation at individual sites, but have not systematically been investigated [98,99].

Although glycosylation is a common protein modification, lentiviral proteins are particularly heavily glycosylated [100], with carbohydrate moieties accounting for about 50% of the mass of HIV-1 Env [101,102]. Remarkable variation in the number and position of glycan motifs across variants has long been recognized as a defining feature of HIV-1 Env [76,81,82], and evasion from neutralizing antibodies has been proposed as a major function of this ‘glycan shield’ [8]. In the context of the Env trimer, an extensive glycan ‘canopy’ consists of around 90 N-linked carbohydrate moieties that exist in crowded and dispersed configurations, are under-processed compared to host glycans, and form inter-dependent clusters [103,104]. The oligomeric nature and dense packing of glycans of HIV-1 Env restricts its glycan processing in the golgi such that most glycans are oligomannose, but micro-heterogeneity in carbohydrate forms is observed at individual glycan addition sites [103–106]. The glycan array is known to have a strong influence on Env structure and antigenicity, as well as modulating antibody recognition. Indeed, removal of a single glycan site can influence neutralizing antibody recognition at distal locations as well as reducing the overall oligomannose content by more than predicted [104]. Glycosylation of gp120 could also influence the mucosal transmissibility of HIV-1, depending on the clade and cohort examined [64,68,78,79,107,108]. Our findings, combined with those from previous studies, suggest that the use of NXS vs. NXT in HIV-1 Env could be an additional means for the virus to modulate its structure, immunogenicity, and sensitivity to neutralizing antibodies [10,38]. Recovery and characterization of numerous bnAbs from chronically infected individuals has demonstrated that many of these antibodies have the capacity to target glycans directly [56,109–122]. Even bnAbs that do not target glycans directly, such as VRC01, have acquired unique adaptations that allow the antibody to avoid glycan clashes and tolerate Env diversity [123–125]. Further underscoring the importance of Env glycosylation to vaccine development, Env SOSIP trimer immunogens based on clade B JR-FL and clade A BG505 Envs elicited tier 2 neutralizing antibodies, but their activity was restricted at least in part by the fact that the neutralizing antibodies that targeted glycan ‘holes’, where a particular glycan motif is absent [89,90]. Although we did not find that the absence or presence of a specific glycan(s) was associated with the development of breadth in our cohort, it is possible that T/F Envs with fewer NXS sites are less likely to have glycan ‘holes’, and preferentially elicit antibodies with a greater capacity to acquire breadth in response to Env diversification. Thus, as stated by [126], glycans have ‘enormous relevance’ to HIV-1 vaccine design. It is therefore notable that a potential marker for efficiency of Env glycosylation has emerged in this study, and in a distinct study by [38], as an early clade-specific signal that may contribute to the development of neutralization breadth.

Currently, a substantial effort is being devoted to designing and testing novel HIV-1 Env immunogens that preserve features of the native trimer and/or are derived from patients who developed broadly neutralizing antibody activity [89,127–133]. These Env immunogens could be readily varied in terms of glycosylation and immunotype diversity, and assessed experimentally, whereas it is less clear how to incorporate sustained viral replication, continuing Env diversity, genotypic host features, and other correlates of breadth via a vaccination protocol. Our results are encouraging for vaccine design because they suggest that modulating Env glycosylation in a clade-specific manner could produce immunogens that are even better suited to elicit neutralizing antibodies with the potential to transition to heterologous breadth. Then, if one can develop immunotype mimics based on diversification of the hyper-variable domains, antibody lineages may be able to develop tolerance for diversity and glycans, and acquire heterologous neutralizing activity to relevant targets. On the other hand, Env diversity presented in the absence of the neutralizing antibody response that provided the selective pressure may be unable to fully recapitulate the evolution of bnAb in immunized animal models [134,135]. However, the strategic selection and presentation of the priming Env appears to be of vital importance, as we have shown for SIV vaccination in nonhuman primate studies [136].

It is important to consider that, during HIV-1 infection, neutralizing antibodies tend to have limited specificities, including the CD4 binding site, V1V2, and the base of the V3 loop, that differ between individuals [10–12,15–17,27,137]. It is unclear why some individuals, such as R66M studied here, produce antibodies with limited neutralizing capacity against the autologous T/F Env while others, such as Z1800M, produce potent neutralizing antibodies within the same time frame. It is possible that, in addition to differences in subtype, the higher NXS to NXT ratio of the R66M T/F Env compared to the Z1800M T/F Env could also have initiated this disparity. Our findings highlight a void in our understanding of the importance of the immunogenicity and antigenicity of the T/F Env, and the early anti-Env antibody landscape, in terms of germline activation, clonality, and binding affinity, on the ability to transition from strain-specific to heterologous neutralization. Broadly neutralizing antibody lineages, such as VRC01 or CAP256, do not develop in isolation; they stem from autologous neutralizing antibodies in an environment that includes competition and influence by a milieu of other B cells and antibodies, occurring in the presence of ongoing viral diversity [27,138]. Current studies have begun to bridge the gap between strain-specific and broadly neutralizing antibodies by focusing on the longitudinal evolution of an individual bnAb lineage [24–27,138]. Others have developed strategies to design Env antigens that engage specific B-cell germline lineages with known potential to evolve into a broadly neutralizing antibody [139,140]. However, even this strategically designed Env immunogen engaged other germlines, in addition to the germline of interest, suggesting that the complexity of the human immune system will be a formidable obstacle to reproducing the activation and maturation of individual bnAb lineages [139]. Taken together, our observations provide new insight into the importance of T/F Env glycosylation, autologous neutralizing antibodies, diversification in gp120, and viral escape in setting the course to neutralization breadth. With the successful elicitation of tier 2 autologous neutralizing antibodies using trimer-based immunogens [89,90], additional promising Env immunogens on the horizon, and a more complete understanding of glycosylation in the context of several genetically diverse Env trimers [103,104], it may be possible to optimize the glycosylation of these reagents so that they preferentially elicit neutralizing antibodies with the potential to acquire heterologous neutralizing capacity. Whether necessary cycles of antibody-virus co-evolution, and other factors, can then be reproduced in the absence of infection to augment this process will remain to be determined.

## Materials and Methods

### Ethics statement

The ZEHRP, PSF, and IAVI Protocol C participants were selected based on rapid screening of adults with recent history of HIV exposure in Rwanda and Zambia. After obtaining written informed consent, blood samples were collected from HIV-1 infected participants longitudinally. The procedures for written informed consent and research activities were approved by institutional review boards at all collaborating clinical research centers, with further compliance to human experimentation guidelines set forth by the United States Department of Health and Human Services. The study was reviewed and approved by the Republic of Rwanda National Ethics Committee, Emory University Institutional Review Board, and the University of Zambia Research Ethics Committee.

### Study population

The 21 HIV-1 infected individuals studied were identified shortly after a transmission event through their enrollment in two HIV-discordant couple cohorts (see Fig 1). Zambian participants were from the Zambia-Emory HIV Research Project (ZEHRP, established in 1994 by Dr. Susan Allen) in Lusaka and the Rwandan participants were from Projet San Francisco (PSF, established in 1986 by Dr. Susan Allen) in Kigali. Together, these two sites accounted for roughly 43% of participants in the larger IAVI study [36]. These projects were originally designed as ‘couples voluntary counseling and testing’ clinics and have been described previously [64,141]. ZEHRP and PSF are also part of the Rwanda Zambia HIV Research Group at Emory University (RZHRG; http://www.rzhrg.org). More recently Protocol C, a uniform vaccine-preparedness study developed and implemented by the International AIDS Vaccine Initiative (IAVI; http://www.iavi.org), was initiated and carried out at multiple sites in Africa, including ZEHRP and PSF [80].

The 21 individuals studied here were selected prior to the initiation of the larger IAVI study. The subjects were chosen based on three factors: a p24 antigen positive test (i.e. infection detected in the early Fiebig stages); the availability of early longitudinal plasma samples; and for Protocol C participants, the availability of viable PBMC collected during early infection (for studies that are not included in these analyses). Z185F, Z221M, Z205F, Z153M, and Z201M were enrolled in ZEHRP prior to Protocol C. The remaining 16 subjects were enrolled in Protocol C at the time of sampling. Twelve of 16 the Protocol C participants were analyzed in both the present study and in the larger IAVI study (see Fig 1; note that while there are 13 Parent Study participants with PC codes listed in the second column, Z1022M/PC138 was not analyzed in the larger study and thus has no breadth score in the fifth column) [36].

The procedures for written informed consent and research activities were approved by institutional review boards at all collaborating clinical research centers, with further compliance to human experimentation guidelines set forth by the United States Department of Health and Human Services. Plasma viral load determinations were underwritten by IAVI and performed at Contract Lab Services (CLS) in South Africa using an Abbott m2000 system. The typical range of detection was between 160 and 4×10^7^ RNA copies/ml. HLA genotyping was performed using a combination of PCR-based techniques as described previously [60,142].

### PCR amplification and cloning of HIV-1 *env* genes

For 16 of 21 individuals, cDNA synthesis and 384-well single genome PCR amplification (SGA) was performed essentially as described in [61] S3 Fig. Briefly, RNA was extracted from cryopreserved patient plasma samples using the QIAmp viral RNA, and reverse transcription was performed using the SuperScript III kit (Invitrogen) with reverse primer OFM19 (5’- GCACTCAAGGCAAGCTTTATTGAGGCTTA-3’). cDNA was diluted to result in <30% positive wells for SGA. First round PCR was performed in a 15 μL volume using the Phusion Hotstart II High Fidelity DNA Polymerase (Thermo Scientific) with forward primer Vif1 (5’-GGGTTTATTACAGGGACAGCAGAG-3’) and OFM19. Cycling conditions were 98°C for 2 min; 10 cycles of 95°C for 15 s, 54°C for 60 s, and 68°C for 4 min; 25 cycles of 95°C for 15 s, 54°C for 60 s, and 68°C for 4 min, adding 5 s to the extension per cycle; 72°C for 30 min; and 4°C hold. Second round PCR was performed with the same enzyme in a 10 μL volume with 1 μL of the first round of PCR and EnvA-TOPO (5’-CACCGCCTTAGGCATCTCCTATGGCAGGAAGAA-3’) and EnvN (5’-CTGTCAATCAGGGAAGTAGCCTTGTGT-3’). Cycling conditions were 95°C for 2 min; 30 cycles of 95°C for 15 s, 54°C for 60 s, and 72°C for 2.5 min; 72°C for 10 min; and 4°C hold. PCR amplicons were purified using Qiagen PCR Clean-Up Kit. For Z201M, Z205F, Z221M, Z185F, and Z153M, full-length *env* genes were amplified previously using 96-well SGA from plasma and PBMC, as described [9]. PCR amplified *env* genes plus flanking sequences were T/A-cloned into one of several pCR3.1-based expression vectors as described [9,11,61].

### Sequence analyses of HIV-1 *env* genes

On average, 13 SGA PCR amplicons per patient (range 5 to 31) per time-point were sequenced with Beckman Coulter Genomics using the following primers: For13 (5’- GAGAAAGAGCAGAAGACAGTGG-3’); For15 (5’-CAGCACAGTACAATGTACACATGGAA-3’); For17 (5’- AGCAGCAGGAAGCACTATGGGCGC-3’); For19 (5’-GGAACCTGTGCCTCTTCAGCTACC-3’); and Rev14 (5’-ACCATGTTATTTTTCCACATGTTAAA-3’); Rev16 (5’-ATGGGAGGGGCATACATTGCT-3’); Rev17 (5’- CCTGGAGCTGTTTAATGCCCCAGAC-3’); and Rev19 (5’-ACTTTTTGACCACTTGCCACCCAT-3’). Sequencher v5 was used to generate nucleotide sequence contigs, and sequences with evidence of mixed peaks were omitted from the analysis. Additionally, previously reported sequences were utilized for patients Z205F, Z185F, Z201M, Z221M, Z153M, and R880F [9–12,143,144]. Geneious v6.1.7 was used to align and translate nucleotide sequences. Amino acid alignments were exported from Geneious in FASTA format and used to generate Highlighter plots (http://www.hiv.lanl.gov/content/sequence/HIGHLIGHT/highlighter_top.html), to define Env features (http://www.hiv.lanl.gov/content/sequence/GLYCOSITE/glycosite.html) and (http://www.hiv.lanl.gov/content/sequence/VAR_REG_CHAR/index.html), and perform Sequence Harmony comparisons (http://www.ibi.vu.nl/programs/shmrwww/). Subtype reference Env nucleotide sequences were obtained from the Los Alamos Sequence Database (http://www.hiv.lanl.gov/content/sequence/NEWALIGN/align.html). A Neighbor-joining nucleotide phylogenetic tree was generated in Geneious v6.1.7. HIV-1 subtyping was performed with REGA HIV-1 Automated Subtyping Tool (http://dbpartners.stanford.edu:8080/RegaSubtyping/stanford-hiv/typingtool/) [67]. The days since the most common recent ancestor (MRCA) are presented in Fig 1, and were determined by analyzing the T/F Env sequences for each subject in the Poisson Fitter v2 tool from the Los Alamos HIV Database (http://www.hiv.lanl.gov/content/sequence/POISSON_FITTER/pfitter.html) [58,145]. The estimated days since infection values, also presented in Fig 1, were calculated using the methods of [59]. The T/F *env* nucleotide sequences have been submitted under Genbank accession numbers KX983471-KX983929.

For calculation of IDI scores, Sequence Harmony comparisons were used to identify amino acid positions significantly different between the T/F and 4–8 month sequences (Z-score < -3). For significant positions, points were given as follows: 0.5 points: conservative changes; 1 point: non-conservative changes, deletions, insertions, purifying selection; 1 point: shift, deletion, or insertion of glycosylation motif. Multiple changes at significant positions were given cumulative points. Sum of points at significant positions were multiplied by the absolute value of the Z-score at corresponding position to weight scores by statistical significance. Final IDI for each patient was calculated by adding all points for all positions. For V2-V4-V5 scores, significant positions were restricted to these locations, as identified by LANL HIV Variable Region Characterization tool.

### Neutralization assay

A global panel of 12 HIV-1 Env reference clones developed by deCamp *et al*. was obtained though the NIH AIDS Reagent Program (Catalog number 12670) [51]. Generation of Env pseudoviruses and performing the TZM-bl neutralization assay has been described previously [9–12,40,61,68,136,144,146–150]. Briefly, Env-expressing plasmids were co-transfected with the HIV-1 SG3ΔEnv proviral backbone into 293T cells using Fugene HD (Promega), and pseudovirus stocks were collected 48h post-transfection, clarified by centrifugation, and frozen at -80°C. In all cases except one, plasma was used to evaluate heterologous and autologous neutralization. Because R66M began antiretroviral therapy approximately 1.25 years post-infection, IgG antibodies were purified from 36-month plasma using a GE Healthcare Life Sciences Ab SpinTrap, according to manufacturer instructions (GE 28-4083-47). The concentration of the purified IgG was determined by ELISA, and was used in place of plasma in neutralization assays. Five-fold serial dilutions of heat-inactivated plasma samples or purified IgG were assayed for their inhibitory potential against the Env pseudoviruses using the TZM-bl indicator cell line, with luciferase as the readout. At 48 hours post-infection, cells were lysed and luciferase activity was measured using a BioTek Cytation 3 imaging reader with Gen5 v2.07 Software. The average background luminescence from a series of uninfected wells was subtracted from each experimental well. Assays were run in duplicate and repeated independently at least twice.

### Statistical analyses

All graphs were generated in Prism v6.0. Virus infectivity curves were generated and Area Under the Curve was calculated in Prism and used for comparisons of neutralization activity. Neutralization IC50 titers were also calculated in Prism from the virus infectivity curve using log transformation of x-values, normalization of y-values, and linear regression of dose-response inhibition with variable slope. A Mann-Whitney test or Wilcoxon matched pairs analysis was used to compare two groups, while a Kruskal-Wallis test was used for to compare more than two groups. A non-parametric Spearman’s test was used to assess correlations between variables in Prism v6.0. P values less than 0.05 were considered to be significant.

## Supporting Information

S1 Fig Neutralization Area Under the Curve (AUC) and IC50 titers heat maps for plasma from 21 HIV- 1 infected individuals against the 12 Env global reference panel. Five-fold serial dilutions of plasma samples from each individual (RZHRG coded IDs are shown; see Fig 1) were assayed for neutralization against a panel of globally representative tier 2 Env pseudoviruses, as well as a VSV-G pseudotyped negative control. **A)** The AUC was calculated from each infectivity curve and color-coded on a continuous relative scale from the lowest AUC value (0.3, green) to highest (6.3, red) to visualize differences in breadth and potency of neutralization. **B)** The neutralization 50% inhibition (IC50) titer was calculated from each infectivity curve and color-coded on a continuous relative scale from the lowest IC50 titer (20 indicating that an IC50 could not be calculated, green) to the highest IC50 titer (7424, red) to visualize differences in breadth and potency of neutralization. Note that on both heat maps green indicates the most potent neutralization, while red indicates the least potent.
(TIF)

S2 Fig Neutralization breadth ranking was highly correlated with that determined in the larger IAVI study. For 12 individuals that were analyzed by [36] and in the present study, a Spearman Rank correlation test was performed using the maximum breadth score (sMAX, based on plasma from multiple time points) and the neutralization breadth AUC determine here using plasma from approximately 3-years post infection. The rankings were highly inversely correlated (r = -0.79, p = 0.002), indicating that high sMAX scores corresponded to low breadth AUC values. The sMAX values were plotted on the y-axis against the neutralization breadth AUC on the x-axis. IAVI breadth scores at 36 (red) and 42 (blue) months were also significantly correlated with neutralization breadth AUC.
(TIF)

S3 Fig Highligher plots of T/F Env sequences. **(A-E)** Single genome PCR amplification (SGA) derived full-length *env* sequences were isolated from 21 patients near the time of infection (median 28 estimated days after infection, range 22 to 65 days). Translated amino acid sequences were aligned, and the LANL Highlighter tool was used to illustrate sequence variation using ticks colored as indicated in the legend.
(PDF)

S4 Fig Phylogenetic tree and recombination breakpoints. A selection of 15 Subtype Reference Sequences was downloaded from the LANL sequence database and were aligned with the consensus of each set of patient derived T/F Env sequences. The resulting neighbor-joining phylogenetic tree, shown in **(A)**, illustrates Zambian derived Envs clustering with the Subtype C reference sequences, while the majority of Rwandan sequences cluster with Subtype A1. The exceptions to this, R1142F **(B)** and R66M **(C)**, were analyzed via the REGA HIV-1 Subtyping Tool to identify contributing components of these recombinant Envs.
(TIF)

S5 Fig NXS and NXT glycan motifs within T/F Env gp120 sequences. **(A)** An amino acid alignment of 21 T/F Env gp120 sequences was analyzed for glycosylation sequon and position using LANL N-GlycoSite (https://www.hiv.lanl.gov/content/sequence/GLYCOSITE/glycosite.html). Numbering includes amino acid position according to the T/F Env alignment, and relative to HXB2 in parentheses. Variable regions are highlighted above the position numbers. The number of NXS, NXT, and total glycans, and NXS: NXT ratio is included for each sequence. **(B)** To determine if NXS:NXT ratios were driven by differences in a specific region of Env, data was divided into the top 10 neutralizers (#1–10) and bottom 11 neutralizers (#11–21) based on breadth ranking. NXS:NXT ratios were calculated for V1, V2, C2, V3, C3, V4, C4, and V5 regions of gp120 by dividing the number of NXS sites in that region by the number of NXT sites for each group of T/F Envs. The T/F Envs from the bottom 11 neutralizers had higher NXS:NXT ratios in V1, V2, C2, C3, V4, and C4, highlighted in yellow, but not in V3 and V5.
(TIF)

S6 Fig Amino acid alignments of Env for Z1800M and R66M. Env amino acid alignments are shown for Z1800M **(A)** and R66M **(B)**, including sequences from the first time point after infection (estimated to be 29 days for Z1800M and 22 days for R66M, Fig 1) and the longitudinal time point used to calculate the Immunotype Diversity Index score (5-months for Z1800M and R66M). The Env IDI scores for Z1800M and R66M were 263 and 28, respectively (Fig 5). The V2,V4,V5 IDI scores were 232 and 0, respectively (Fig 5). The sequences from the 5-month time point are shaded gray. Yellow highlighting indicates positions that were identified by Sequence Harmony as having a Z-score less than -3. gp120 variable domains are indicated above the sequences.
(PDF)
