# Supplementary figures and images for: Diversification in the HIV-1 Envelope Hyper-variable Domains V2, V4, and V5 and Higher Probability of Transmitted/Founder Envelope Glycosylation Favor the Development of Heterologous Neutralization Breadth
Source: PLoS Pathog. 2016 Nov 16;12(11):e1005989. doi: 10.1371/journal.ppat.1005989 (PMC5112890; doi:10.1371/journal.ppat.1005989)

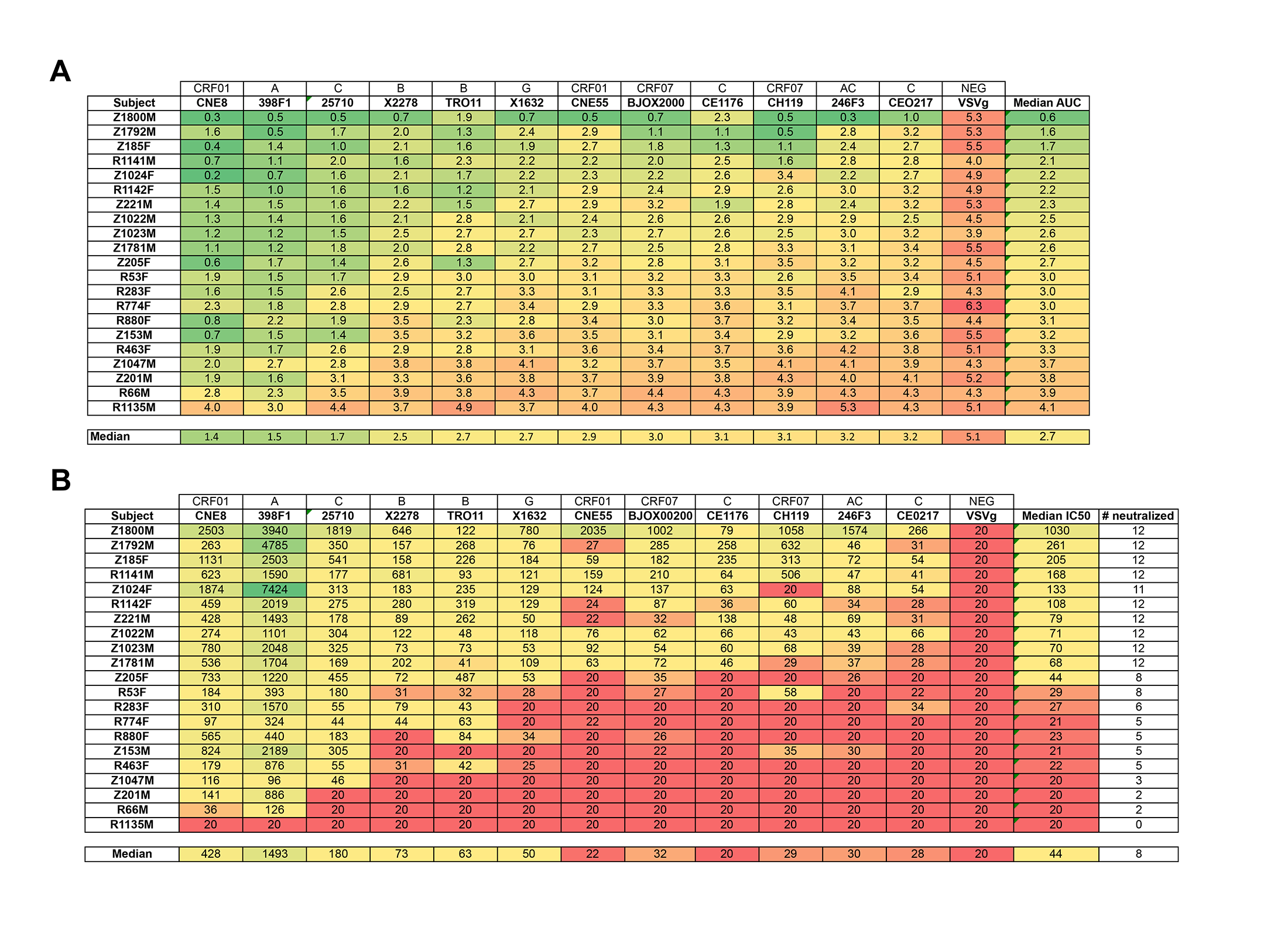

Supplement: S1 Fig — Five-fold serial dilutions of plasma samples from each individual (RZHRG coded IDs are shown; see Fig 1) were assayed for neutralization against a panel of globally representative tier 2 Env pseudoviruses, as well as a VSV-G pseudotyped negative control. A) The AUC was calculated from each infectivity curve and color-coded on a continuous relative scale from the lowest AUC value (0.3, green) to highest (6.3, red) to visualize differences in breadth and potency of neutralization. B) The neutralization 50% inhibition (IC50) titer was calculated from each infectivity curve and color-coded on a continuous relative scale from the lowest IC50 titer (20 indicating that an IC50 could not be calculated, green) to the highest IC50 titer (7424, red) to visualize differences in breadth and potency of neutralization. Note that on both heat maps green indicates the most potent neutralization, while red indicates the least potent. (TIF) [file ppat.1005989.s001.tif]

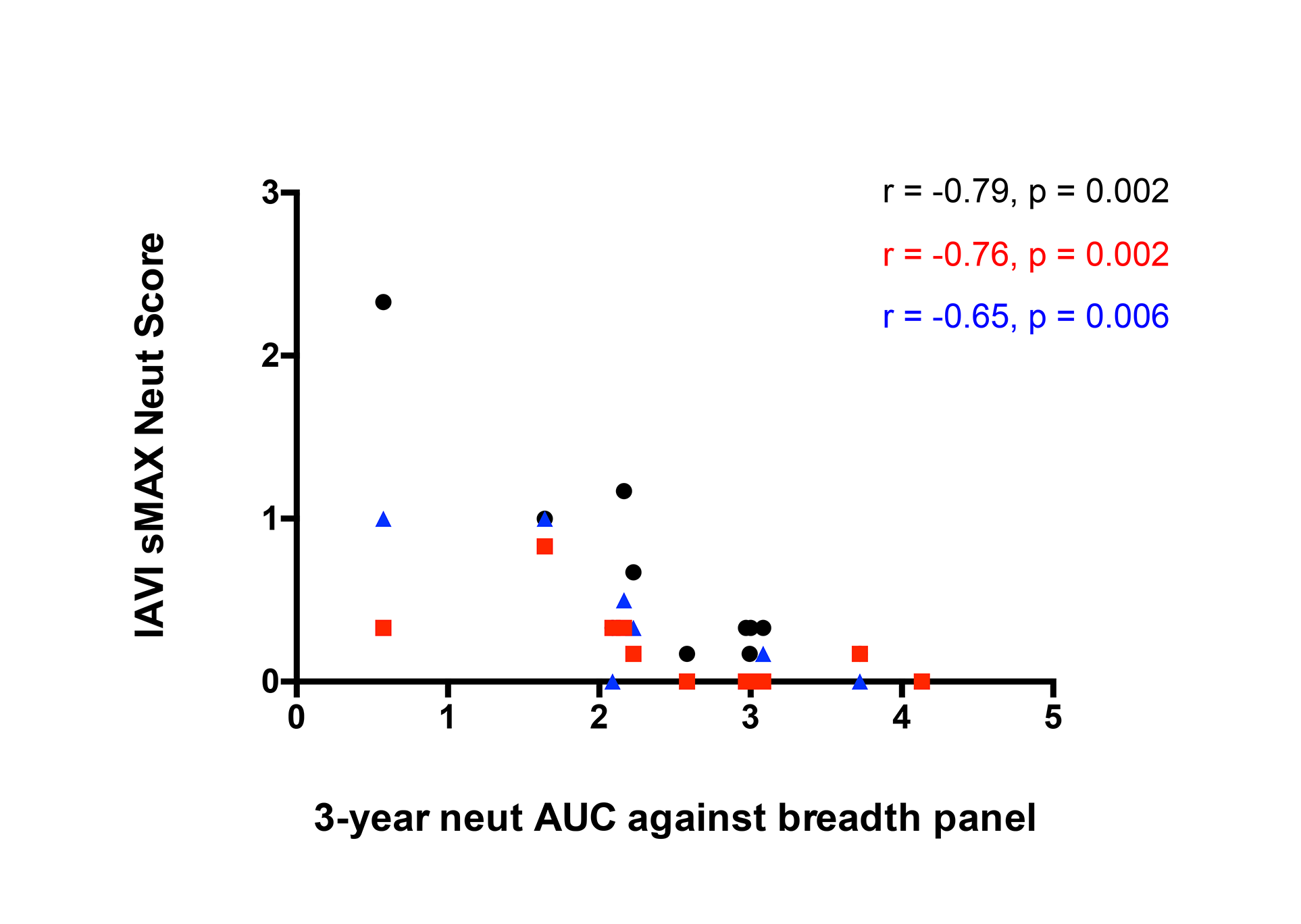

Supplement: S2 Fig — For 12 individuals that were analyzed by [36] and in the present study, a Spearman Rank correlation test was performed using the maximum breadth score (sMAX, based on plasma from multiple time points) and the neutralization breadth AUC determine here using plasma from approximately 3-years post infection. The rankings were highly inversely correlated (r = -0.79, p = 0.002), indicating that high sMAX scores corresponded to low breadth AUC values. The sMAX values were plotted on the y-axis against the neutralization breadth AUC on the x-axis. IAVI breadth scores at 36 (red) and 42 (blue) months were also significantly correlated with neutralization breadth AUC. (TIF) [file ppat.1005989.s002.tif]

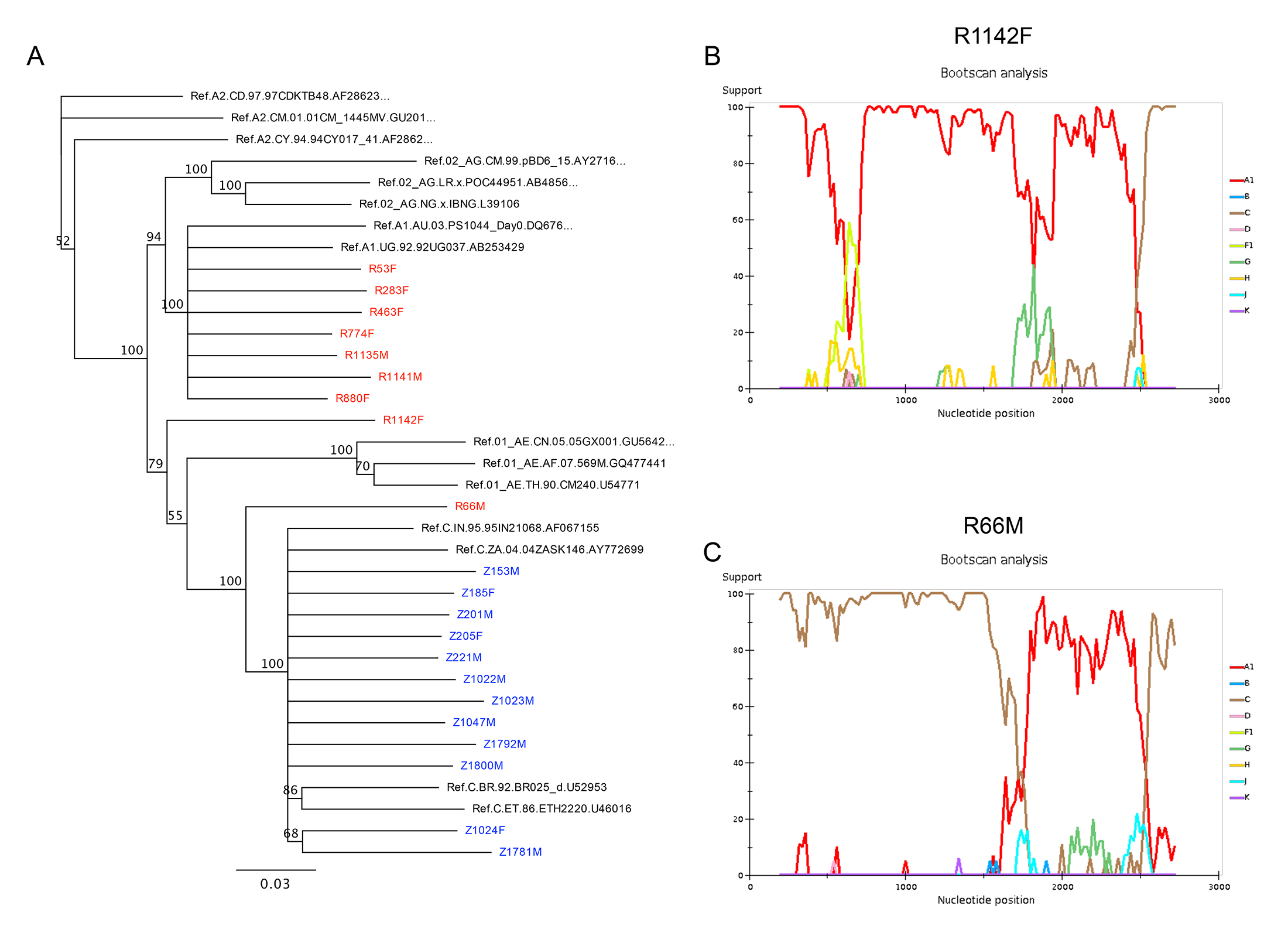

Supplement: S4 Fig — A selection of 15 Subtype Reference Sequences was downloaded from the LANL sequence database and were aligned with the consensus of each set of patient derived T/F Env sequences. The resulting neighbor-joining phylogenetic tree, shown in (A), illustrates Zambian derived Envs clustering with the Subtype C reference sequences, while the majority of Rwandan sequences cluster with Subtype A1. The exceptions to this, R1142F (B) and R66M (C), were analyzed via the REGA HIV-1 Subtyping Tool to identify contributing components of these recombinant Envs. (TIF) [file ppat.1005989.s004.tif]

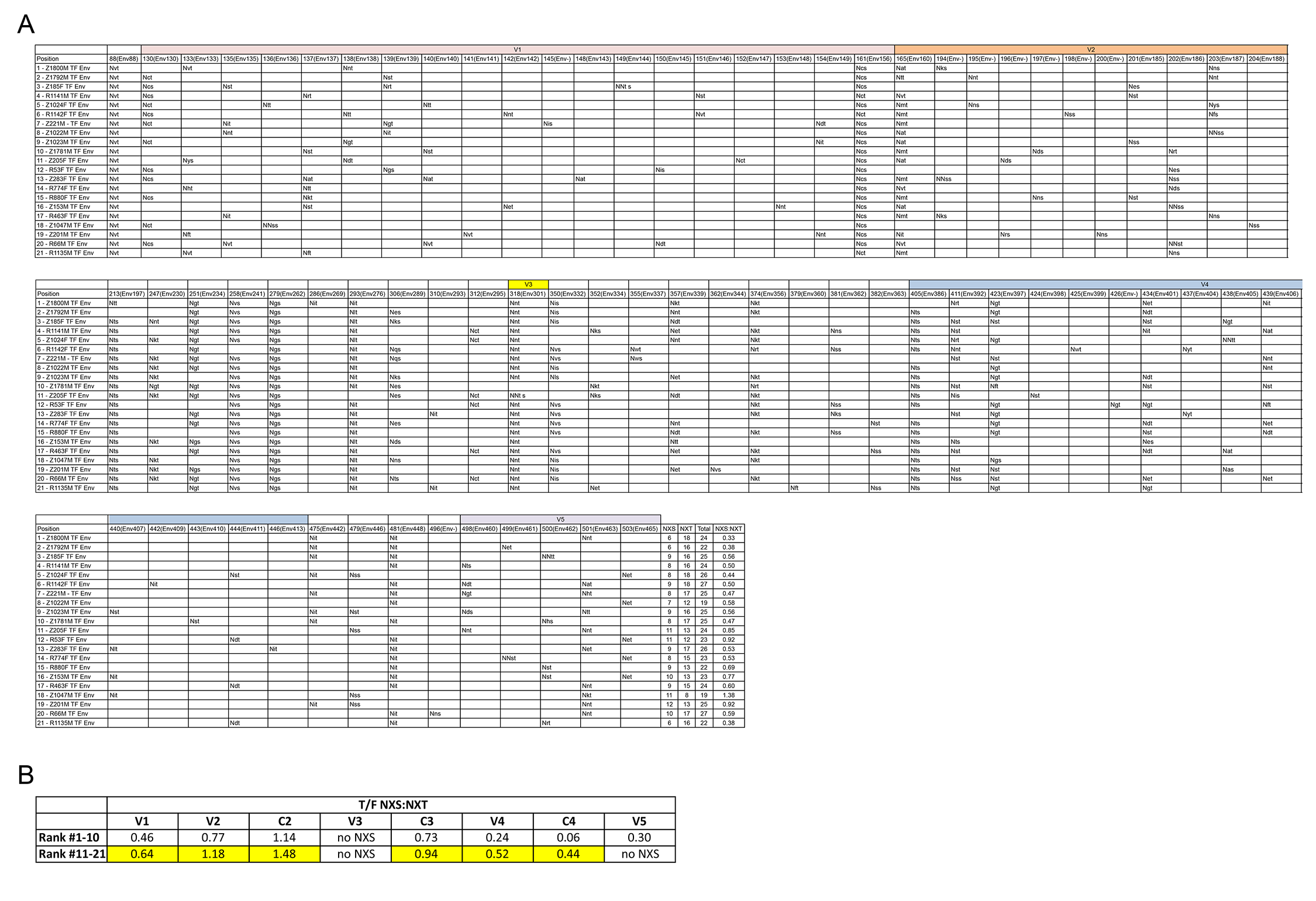

Supplement: S5 Fig — (A) An amino acid alignment of 21 T/F Env gp120 sequences was analyzed for glycosylation sequon and position using LANL N-GlycoSite (https://www.hiv.lanl.gov/content/sequence/GLYCOSITE/glycosite.html). Numbering includes amino acid position according to the T/F Env alignment, and relative to HXB2 in parentheses. Variable regions are highlighted above the position numbers. The number of NXS, NXT, and total glycans, and NXS: NXT ratio is included for each sequence. (B) To determine if NXS:NXT ratios were driven by differences in a specific region of Env, data was divided into the top 10 neutralizers (#1–10) and bottom 11 neutralizers (#11–21) based on breadth ranking. NXS:NXT ratios were calculated for V1, V2, C2, V3, C3, V4, C4, and V5 regions of gp120 by dividing the number of NXS sites in that region by the number of NXT sites for each group of T/F Envs. The T/F Envs from the bottom 11 neutralizers had higher NXS:NXT ratios in V1, V2, C2, C3, V4, and C4, highlighted in yellow, but not in V3 and V5. (TIF) [file ppat.1005989.s005.tif]
