# Supplementary material for: Diversification in the HIV-1 Envelope Hyper-variable Domains V2, V4, and V5 and Higher Probability of Transmitted/Founder Envelope Glycosylation Favor the Development of Heterologous Neutralization Breadth
Source: PLoS Pathog. 2016 Nov 16;12(11):e1005989. doi: 10.1371/journal.ppat.1005989 (PMC5112890; doi:10.1371/journal.ppat.1005989)
Supplement: S3 Fig — (A-E) Single genome PCR amplification (SGA) derived full-length env sequences were isolated from 21 patients near the time of infection (median 28 estimated days after infection, range 22 to 65 days). Translated amino acid sequences were aligned, and the LANL Highlighter tool was used to illustrate sequence variation using ticks colored as indicated in the legend. (PDF) [file ppat.1005989.s003.pdf]

A

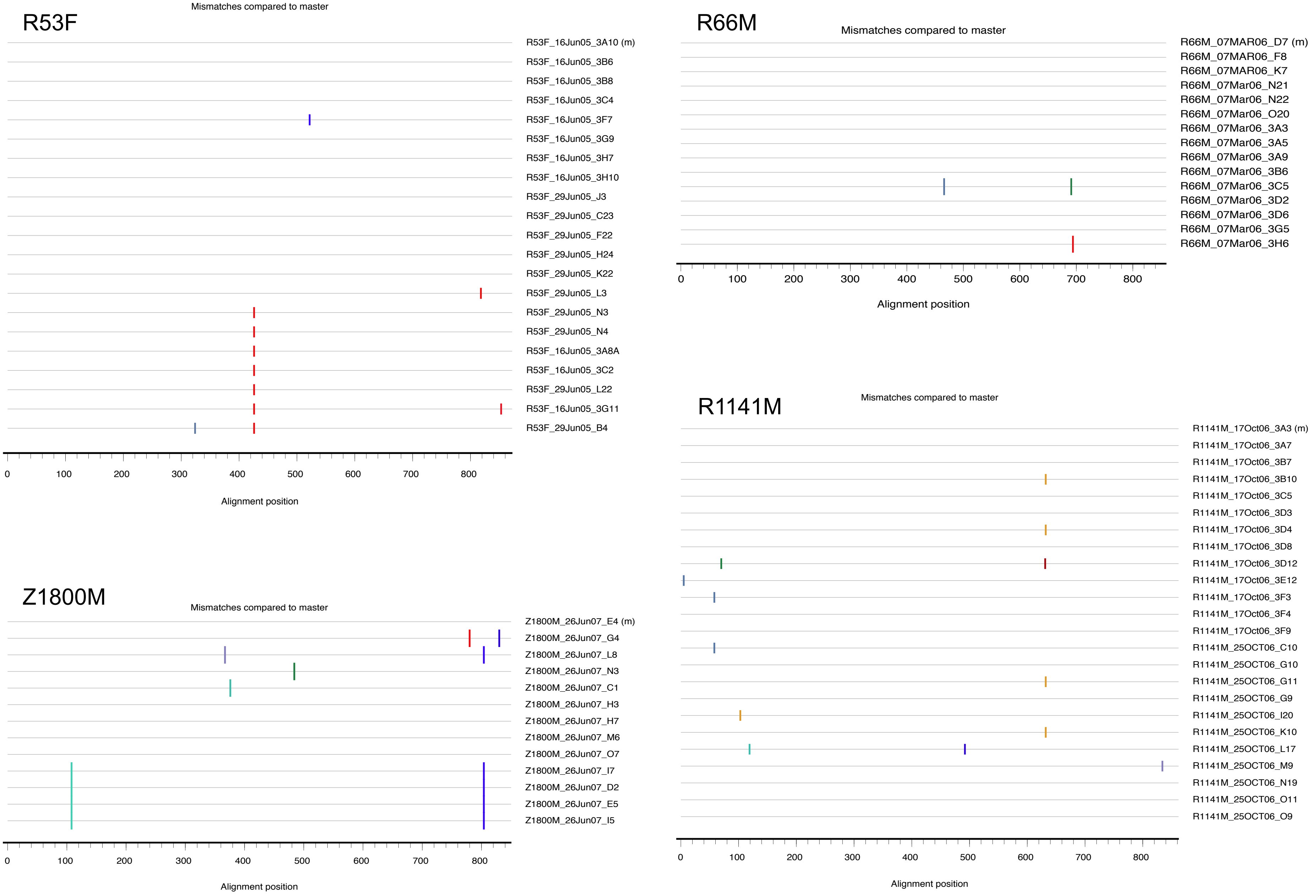
R53F
Mismatches compared to master
R53F_16Jun05_3A10 (m)
R53F_16Jun05_3B6
R53F_16Jun05_3B8
R53F_16Jun05_3C4
R53F_16Jun05_3F7
R53F_16Jun05_3G9
R53F_16Jun05_3H7
R53F_16Jun05_3H10
R53F_29Jun05_J3
R53F_29Jun05_C23
R53F_29Jun05_F22
R53F_29Jun05_H24
R53F_29Jun05_K22
R53F_29Jun05_L3
R53F_29Jun05_N3
R53F_29Jun05_N4
R53F_16Jun05_3A8A
R53F_16Jun05_3C2
R53F_29Jun05_L22
R53F_16Jun05_3G11
R53F_29Jun05_B4
0
100
200
300
400
500
600
700
800
Alignment position
R66M
Mismatches compared to master
R66M_07MAR06_D7 (m)
R66M_07MAR06_F8
R66M_07MAR06_K7
R66M_07Mar06_N21
R66M_07Mar06_N22
R66M_07Mar06_O20
R66M_07Mar06_3A3
R66M_07Mar06_3A5
R66M_07Mar06_3A9
R66M_07Mar06_3B6
R66M_07Mar06_3C5
R66M_07Mar06_3D2
R66M_07Mar06_3D6
R66M_07Mar06_3G5
R66M_07Mar06_3H6
0
100
200
300
400
500
600
700
800
Alignment position
Z1800M
Mismatches compared to master
Z1800M_26Jun07_E4 (m)
Z1800M_26Jun07_G4
Z1800M_26Jun07_L8
Z1800M_26Jun07_N3
Z1800M_26Jun07_C1
Z1800M_26Jun07_H3
Z1800M_26Jun07_H7
Z1800M_26Jun07_M6
Z1800M_26Jun07_O7
Z1800M_26Jun07_I7
Z1800M_26Jun07_D2
Z1800M_26Jun07_E5
Z1800M_26Jun07_I5
0
100
200
300
400
500
600
700
800
Alignment position
R1141M
Mismatches compared to master
R1141M_17Oct06_3A3 (m)
R1141M_17Oct06_3A7
R1141M_17Oct06_3B7
R1141M_17Oct06_3B10
R1141M_17Oct06_3C5
R1141M_17Oct06_3D3
R1141M_17Oct06_3D4
R1141M_17Oct06_3D8
R1141M_17Oct06_3D12
R1141M_17Oct06_3E12
R1141M_17Oct06_3F3
R1141M_17Oct06_3F4
R1141M_17Oct06_3F9
R1141M_25OCT06_C10
R1141M_25OCT06_G10
R1141M_25OCT06_G11
R1141M_25OCT06_G9
R1141M_25OCT06_I20
R1141M_25OCT06_K10
R1141M_25OCT06_L17
R1141M_25OCT06_M9
R1141M_25OCT06_N19
R1141M_25OCT06_O11
R1141M_25OCT06_O9
0
100
200
300
400
500
600
700
800
Alignment position

B

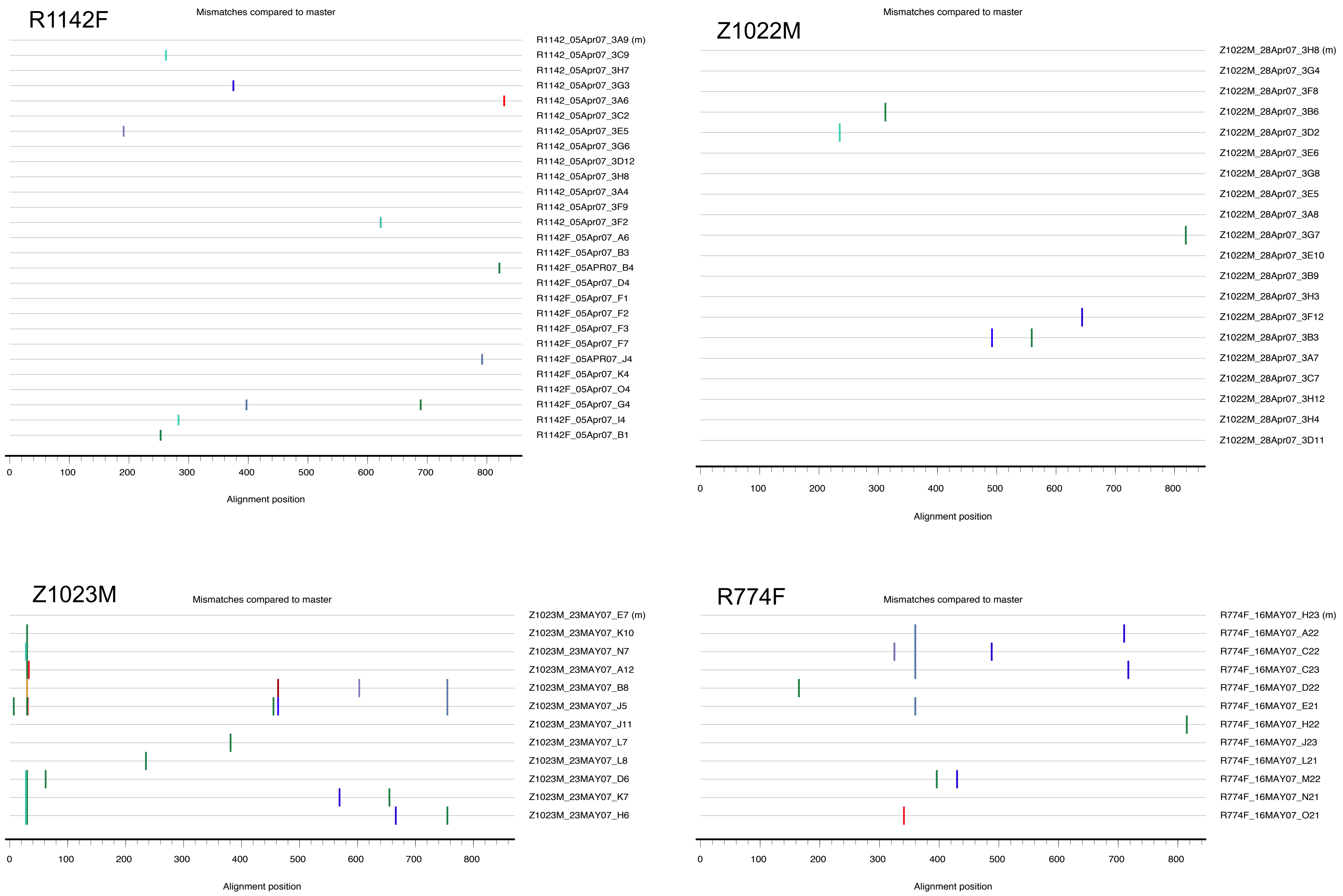
R1142F
Mismatches compared to master
R1142_05Apr07_3A9 (m)
R1142_05Apr07_3C9
R1142_05Apr07_3H7
R1142_05Apr07_3G3
R1142_05Apr07_3A6
R1142_05Apr07_3C2
R1142_05Apr07_3E5
R1142_05Apr07_3G6
R1142_05Apr07_3D12
R1142_05Apr07_3H8
R1142_05Apr07_3A4
R1142_05Apr07_3F9
R1142_05Apr07_3F2
R1142F_05Apr07_A6
R1142F_05Apr07_B3
R1142F_05APR07_B4
R1142F_05Apr07_D4
R1142F_05Apr07_F1
R1142F_05Apr07_F2
R1142F_05Apr07_F3
R1142F_05Apr07_F7
R1142F_05APR07_J4
R1142F_05Apr07_K4
R1142F_05Apr07_O4
R1142F_05Apr07_G4
R1142F_05Apr07_I4
R1142F_05Apr07_B1
0 100 200 300 400 500 600 700 800
Alignment position
Z1022M
Mismatches compared to master
Z1022M_28Apr07_3H8 (m)
Z1022M_28Apr07_3G4
Z1022M_28Apr07_3F8
Z1022M_28Apr07_3B6
Z1022M_28Apr07_3D2
Z1022M_28Apr07_3E6
Z1022M_28Apr07_3G8
Z1022M_28Apr07_3E5
Z1022M_28Apr07_3A8
Z1022M_28Apr07_3G7
Z1022M_28Apr07_3E10
Z1022M_28Apr07_3B9
Z1022M_28Apr07_3H3
Z1022M_28Apr07_3F12
Z1022M_28Apr07_3B3
Z1022M_28Apr07_3A7
Z1022M_28Apr07_3C7
Z1022M_28Apr07_3H12
Z1022M_28Apr07_3H4
Z1022M_28Apr07_3D11
0 100 200 300 400 500 600 700 800
Alignment position
Z1023M
Mismatches compared to master
Z1023M_23MAY07_E7 (m)
Z1023M_23MAY07_K10
Z1023M_23MAY07_N7
Z1023M_23MAY07_A12
Z1023M_23MAY07_B8
Z1023M_23MAY07_J5
Z1023M_23MAY07_J11
Z1023M_23MAY07_L7
Z1023M_23MAY07_L8
Z1023M_23MAY07_D6
Z1023M_23MAY07_K7
Z1023M_23MAY07_H6
0 100 200 300 400 500 600 700 800
Alignment position
R774F
Mismatches compared to master
R774F_16MAY07_H23 (m)
R774F_16MAY07_A22
R774F_16MAY07_C22
R774F_16MAY07_C23
R774F_16MAY07_D22
R774F_16MAY07_E21
R774F_16MAY07_H22
R774F_16MAY07_J23
R774F_16MAY07_L21
R774F_16MAY07_M22
R774F_16MAY07_N21
R774F_16MAY07_O21
0 100 200 300 400 500 600 700 800
Alignment position

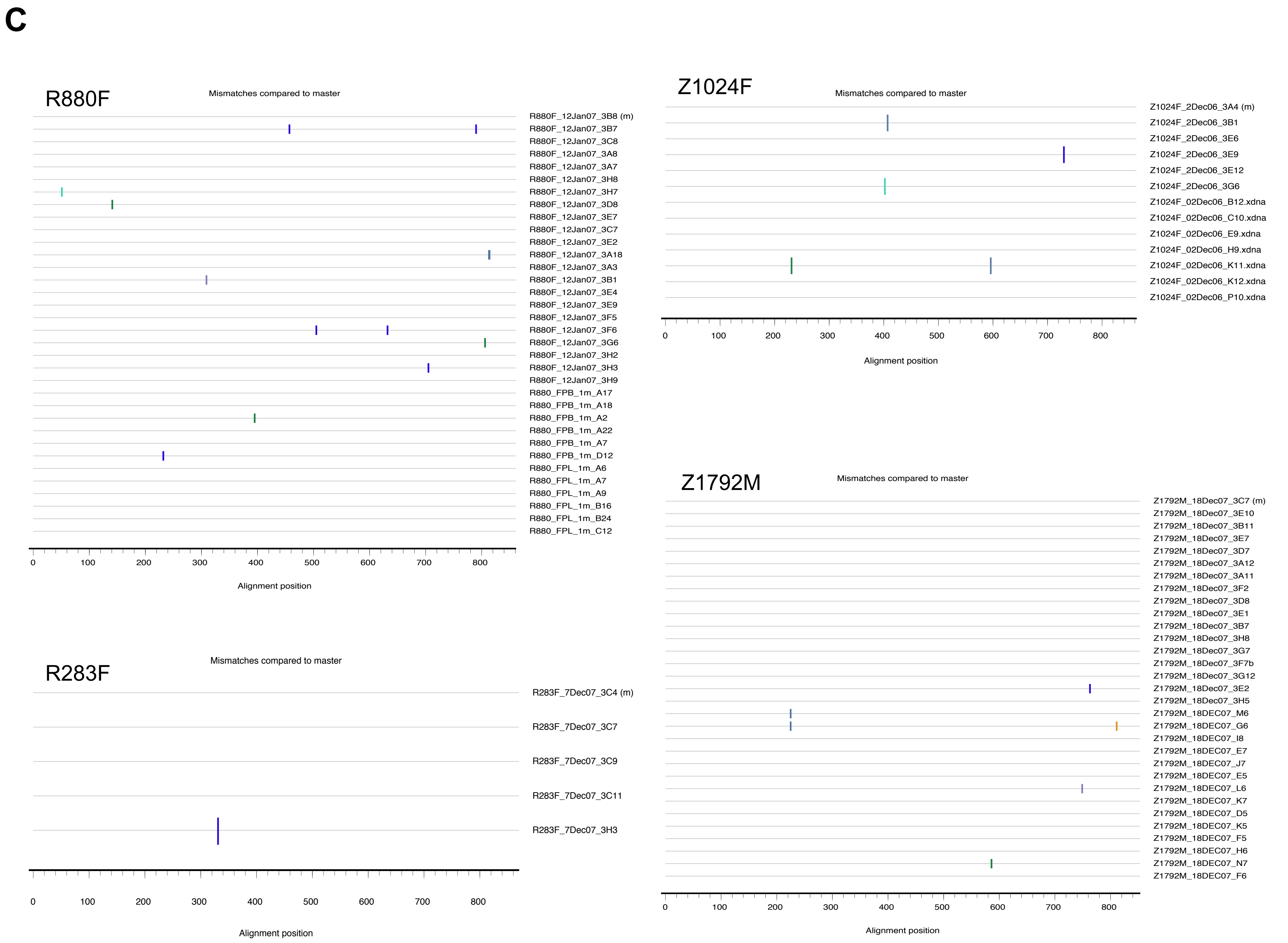
C
R880F
Mismatches compared to master
R880F_12Jan07_3B8 (m)
R880F_12Jan07_3B7
R880F_12Jan07_3C8
R880F_12Jan07_3A8
R880F_12Jan07_3A7
R880F_12Jan07_3H8
R880F_12Jan07_3H7
R880F_12Jan07_3D8
R880F_12Jan07_3E7
R880F_12Jan07_3C7
R880F_12Jan07_3E2
R880F_12Jan07_3A18
R880F_12Jan07_3A3
R880F_12Jan07_3B1
R880F_12Jan07_3E4
R880F_12Jan07_3E9
R880F_12Jan07_3F5
R880F_12Jan07_3F6
R880F_12Jan07_3G6
R880F_12Jan07_3H2
R880F_12Jan07_3H3
R880F_12Jan07_3H9
R880_FPB_1m_A17
R880_FPB_1m_A18
R880_FPB_1m_A2
R880_FPB_1m_A22
R880_FPB_1m_A7
R880_FPB_1m_D12
R880_FPL_1m_A6
R880_FPL_1m_A7
R880_FPL_1m_A9
R880_FPL_1m_B16
R880_FPL_1m_B24
R880_FPL_1m_C12
0 100 200 300 400 500 600 700 800
Alignment position
Z1024F
Mismatches compared to master
Z1024F_2Dec06_3A4 (m)
Z1024F_2Dec06_3B1
Z1024F_2Dec06_3E6
Z1024F_2Dec06_3E9
Z1024F_2Dec06_3E12
Z1024F_2Dec06_3G6
Z1024F_02Dec06_B12.xdna
Z1024F_02Dec06_C10.xdna
Z1024F_02Dec06_E9.xdna
Z1024F_02Dec06_H9.xdna
Z1024F_02Dec06_K11.xdna
Z1024F_02Dec06_K12.xdna
Z1024F_02Dec06_P10.xdna
0 100 200 300 400 500 600 700 800
Alignment position
R283F
Mismatches compared to master
R283F_7Dec07_3C4 (m)
R283F_7Dec07_3C7
R283F_7Dec07_3C9
R283F_7Dec07_3C11
R283F_7Dec07_3H3
0 100 200 300 400 500 600 700 800
Alignment position
Z1792M
Mismatches compared to master
Z1792M_18Dec07_3C7 (m)
Z1792M_18Dec07_3E10
Z1792M_18Dec07_3B11
Z1792M_18Dec07_3E7
Z1792M_18Dec07_3D7
Z1792M_18Dec07_3A12
Z1792M_18Dec07_3A11
Z1792M_18Dec07_3F2
Z1792M_18Dec07_3D8
Z1792M_18Dec07_3E1
Z1792M_18Dec07_3B7
Z1792M_18Dec07_3H8
Z1792M_18Dec07_3G7
Z1792M_18Dec07_3F7b
Z1792M_18Dec07_3G12
Z1792M_18Dec07_3E2
Z1792M_18Dec07_3H5
Z1792M_18DEC07_M6
Z1792M_18DEC07_G6
Z1792M_18DEC07_I8
Z1792M_18DEC07_E7
Z1792M_18DEC07_J7
Z1792M_18DEC07_E5
Z1792M_18DEC07_L6
Z1792M_18DEC07_K7
Z1792M_18DEC07_D5
Z1792M_18DEC07_K5
Z1792M_18DEC07_F5
Z1792M_18DEC07_H6
Z1792M_18DEC07_N7
Z1792M_18DEC07_F6
0 100 200 300 400 500 600 700 800
Alignment position

D

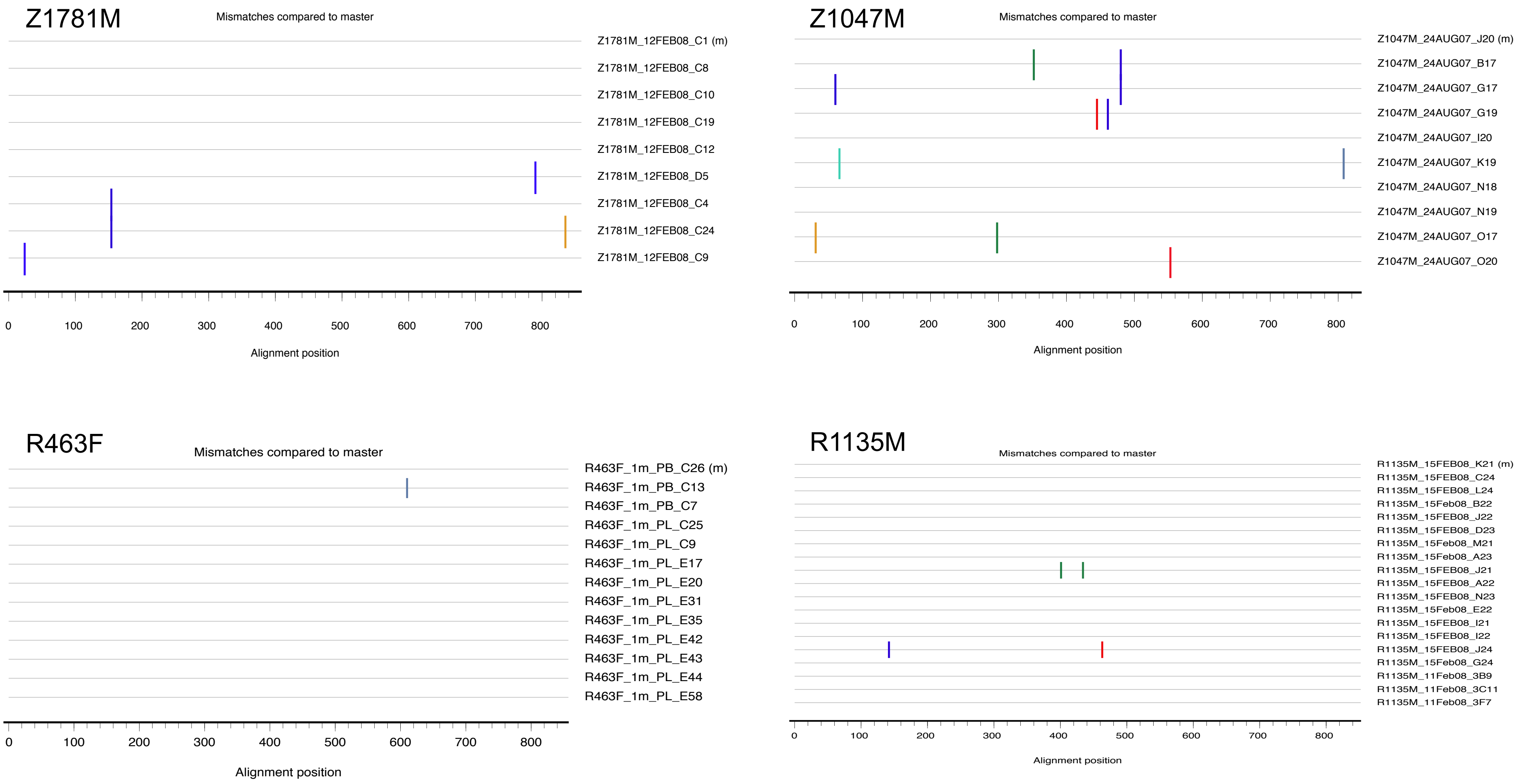

Z1781M
Mismatches compared to master
Z1781M_12FEB08_C1 (m)
Z1781M_12FEB08_C8
Z1781M_12FEB08_C10
Z1781M_12FEB08_C19
Z1781M_12FEB08_C12
Z1781M_12FEB08_D5
Z1781M_12FEB08_C4
Z1781M_12FEB08_C24
Z1781M_12FEB08_C9
0 100 200 300 400 500 600 700 800
Alignment position
Z1047M
Mismatches compared to master
Z1047M_24AUG07_J20 (m)
Z1047M_24AUG07_B17
Z1047M_24AUG07_G17
Z1047M_24AUG07_G19
Z1047M_24AUG07_I20
Z1047M_24AUG07_K19
Z1047M_24AUG07_N18
Z1047M_24AUG07_N19
Z1047M_24AUG07_O17
Z1047M_24AUG07_O20
0 100 200 300 400 500 600 700 800
Alignment position
R463F
Mismatches compared to master
R463F_1m_PB_C26 (m)
R463F_1m_PB_C13
R463F_1m_PB_C7
R463F_1m_PL_C25
R463F_1m_PL_C9
R463F_1m_PL_E17
R463F_1m_PL_E20
R463F_1m_PL_E31
R463F_1m_PL_E35
R463F_1m_PL_E42
R463F_1m_PL_E43
R463F_1m_PL_E44
R463F_1m_PL_E58
0 100 200 300 400 500 600 700 800
Alignment position
R1135M
Mismatches compared to master
R1135M_15FEB08_K21 (m)
R1135M_15FEB08_C24
R1135M_15FEB08_L24
R1135M_15Feb08_B22
R1135M_15FEB08_J22
R1135M_15FEB08_D23
R1135M_15Feb08_M21
R1135M_15Feb08_A23
R1135M_15FEB08_J21
R1135M_15FEB08_A22
R1135M_15FEB08_N23
R1135M_15Feb08_E22
R1135M_15FEB08_I21
R1135M_15FEB08_I22
R1135M_15FEB08_J24
R1135M_15Feb08_G24
R1135M_11Feb08_3B9
R1135M_11Feb08_3C11
R1135M_11Feb08_3F7
0 100 200 300 400 500 600 700 800
Alignment position

E

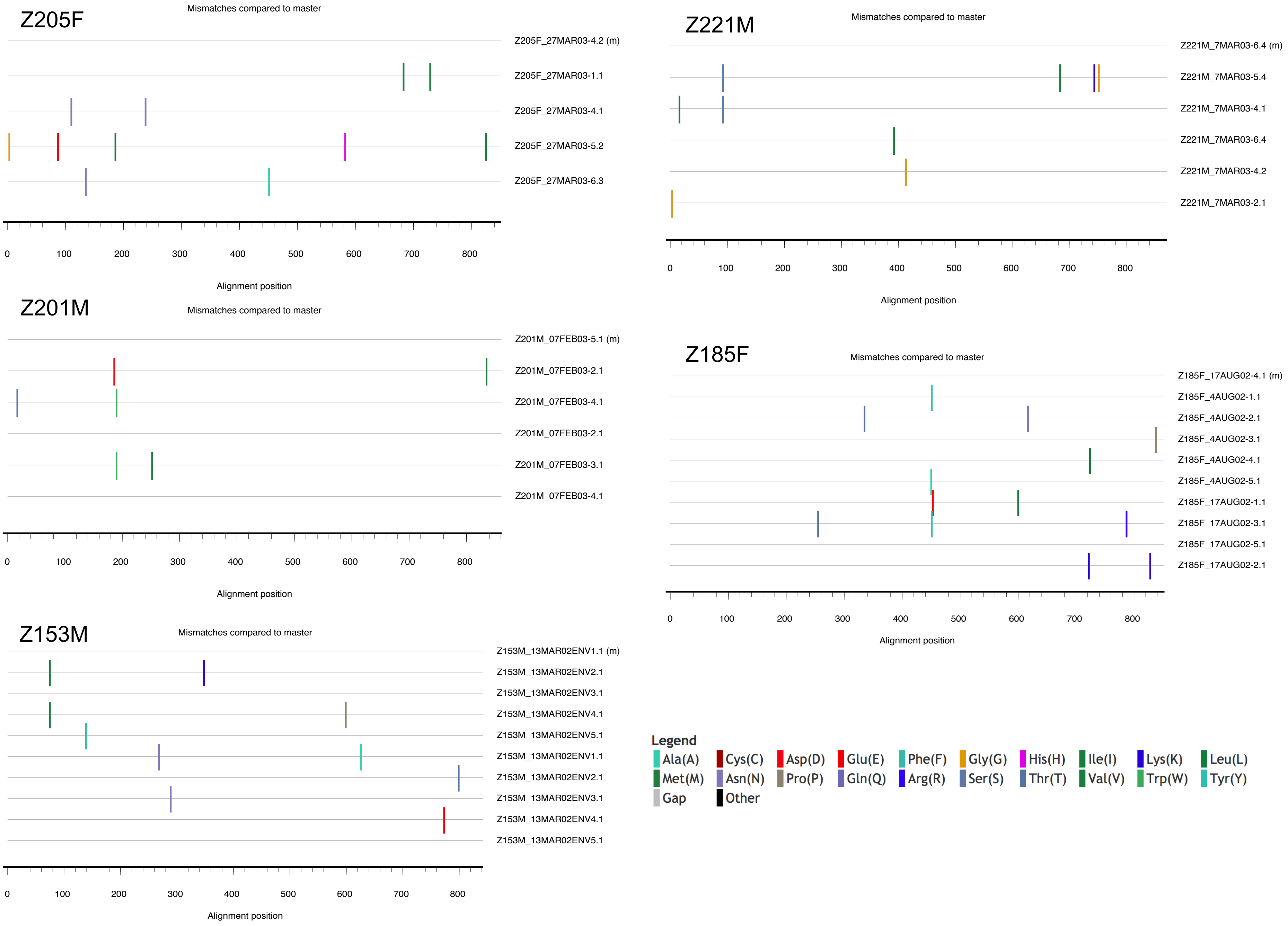

Z205F
Mismatches compared to master
Z205F_27MAR03-4.2 (m)
Z205F_27MAR03-1.1
Z205F_27MAR03-4.1
Z205F_27MAR03-5.2
Z205F_27MAR03-6.3
Alignment position
Z221M
Mismatches compared to master
Z221M_7MAR03-6.4 (m)
Z221M_7MAR03-5.4
Z221M_7MAR03-4.1
Z221M_7MAR03-6.4
Z221M_7MAR03-4.2
Z221M_7MAR03-2.1
Alignment position
Z201M
Mismatches compared to master
Z201M_07FEB03-5.1 (m)
Z201M_07FEB03-2.1
Z201M_07FEB03-4.1
Z201M_07FEB03-2.1
Z201M_07FEB03-3.1
Z201M_07FEB03-4.1
Alignment position
Z185F
Mismatches compared to master
Z185F_17AUG02-4.1 (m)
Z185F_4AUG02-1.1
Z185F_4AUG02-2.1
Z185F_4AUG02-3.1
Z185F_4AUG02-4.1
Z185F_4AUG02-5.1
Z185F_17AUG02-1.1
Z185F_17AUG02-3.1
Z185F_17AUG02-5.1
Z185F_17AUG02-2.1
Alignment position
Z153M
Mismatches compared to master
Z153M_13MAR02ENV1.1 (m)
Z153M_13MAR02ENV2.1
Z153M_13MAR02ENV3.1
Z153M_13MAR02ENV4.1
Z153M_13MAR02ENV5.1
Z153M_13MAR02ENV1.1
Z153M_13MAR02ENV2.1
Z153M_13MAR02ENV3.1
Z153M_13MAR02ENV4.1
Z153M_13MAR02ENV5.1
Alignment position
0 100 200 300 400 500 600 700 800
Legend
Ala(A) Cys(C) Asp(D) Glu(E) Phe(F) Gly(G) His(H) Ile(I) Lys(K) Leu(L)
Met(M) Asn(N) Pro(P) Gln(Q) Arg(R) Ser(S) Thr(T) Val(V) Trp(W) Tyr(Y)
Gap Other
