# Supplementary material for: Diversification in the HIV-1 Envelope Hyper-variable Domains V2, V4, and V5 and Higher Probability of Transmitted/Founder Envelope Glycosylation Favor the Development of Heterologous Neutralization Breadth
Source: PLoS Pathog. 2016 Nov 16;12(11):e1005989. doi: 10.1371/journal.ppat.1005989 (PMC5112890; doi:10.1371/journal.ppat.1005989)
Supplement: S6 Fig — Env amino acid alignments are shown for Z1800M (A) and R66M (B), including sequences from the first time point after infection (estimated to be 29 days for Z1800M and 22 days for R66M, Fig 1) and the longitudinal time point used to calculate the Immunotype Diversity Index score (5-months for Z1800M and R66M). The Env IDI scores for Z1800M and R66M were 263 and 28, respectively (Fig 5). The V2,V4,V5 IDI scores were 232 and 0, respectively (Fig 5). The sequences from the 5-month time point are shaded gray. Yellow highlighting indicates positions that were identified by Sequence Harmony as having a Z-score less than -3. gp120 variable domains are indicated above the sequences. (PDF) [file ppat.1005989.s006.pdf]

A. Z1800M

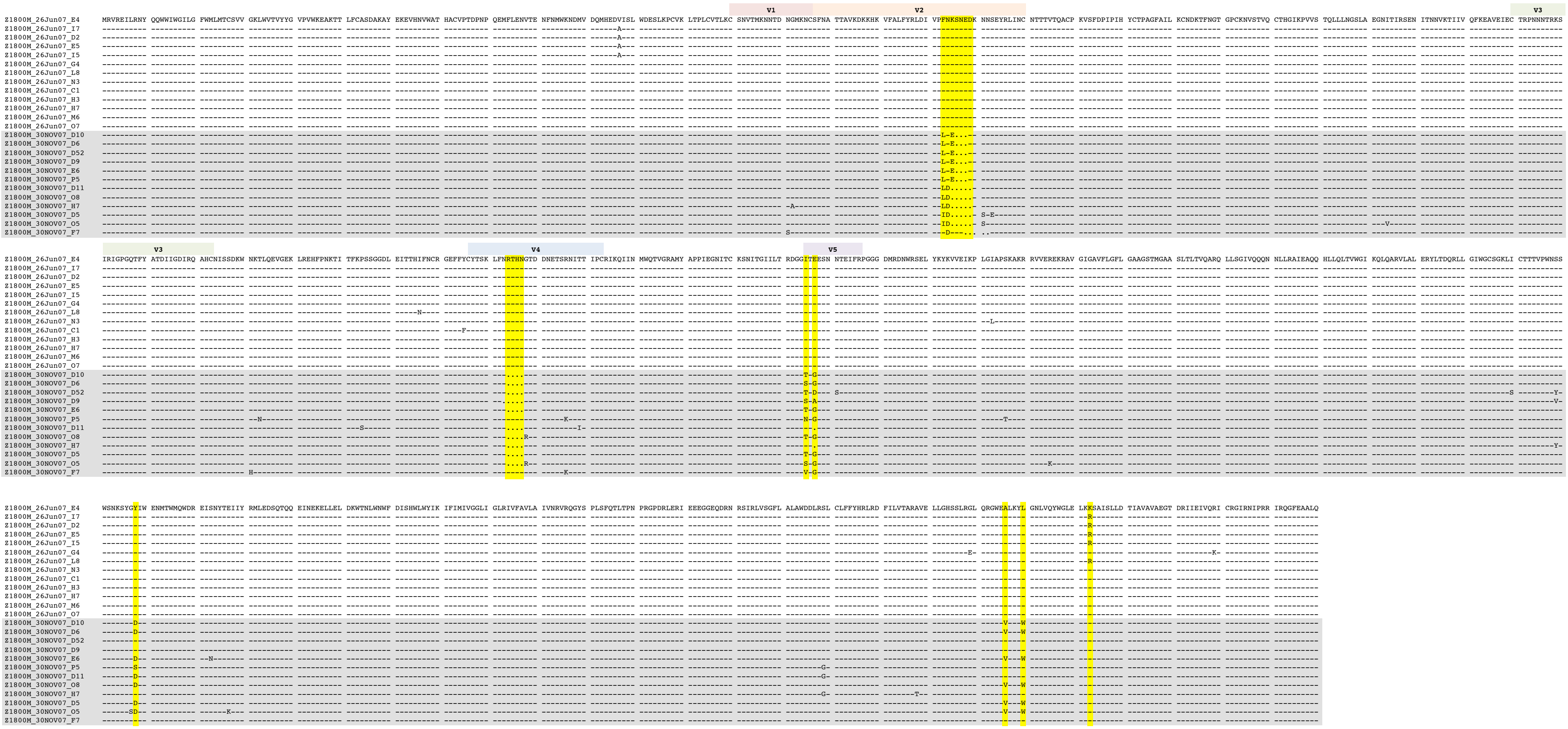

B. R66M

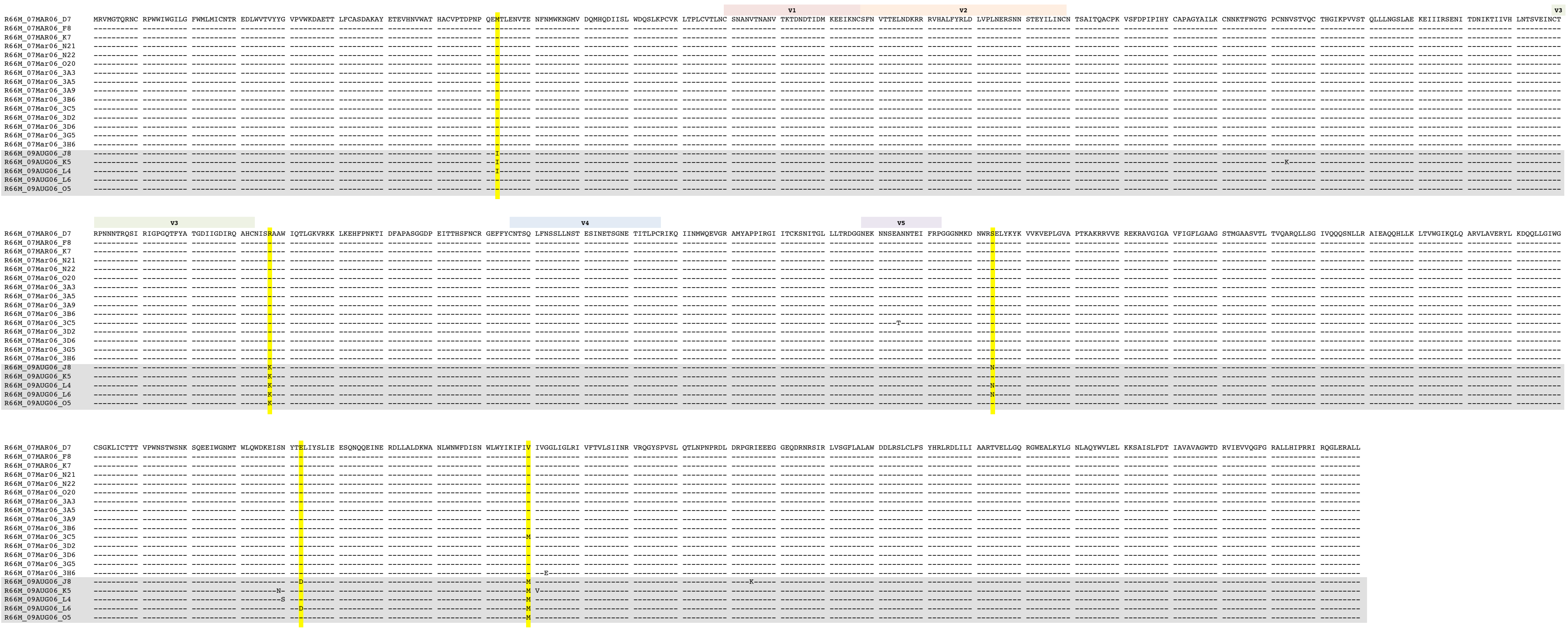
V1
V2
V3
V4
V5
R66M_07MAR06_D7 MRVMGTQRNC RPWWIWGILG FWMLMICNTR EDLWVTVYYG VPVWKDAETT LFCASDAKAY ETEVHNVWAT HACVPTDPNP QEMTLENVTE NFNMWKNGMV DQMHQDIISL WDQSLKPCVK LTPLCVTLNC SNANVTNANV TKTDNDTIDM KEEIKNCSFN VTTELNDKRR RVHALFYRLD LVPLNERSNN STEYILINCN TSAITQACPK VSFDPIPIHY CAPAGYAILK CNNKTFNGTG PCNNVSTVQC THGIKPVVST QLLLNGSLAE KEIIIRSENI TDNIKTIIVH LNTSVEINCT
RPNNNTRQSI RIGPGQTFYA TGDIIGDIRQ AHCNISRAAW IQTLGKVRKK LKEHFPNKTI DFAPASGGDP EITTHSFNCR GEFFYCNTSQ LFNSSLLNST ESINETSGNE TITLPCRIKQ IINMWQEVGR AMYAPPIRGI ITCKSNITGL LLTRDGGNEK NNSEANNTEI FRPGGGNMKD NWRSELYKYK VVKVEPLGVA PTKAKRRVVE REKRAVGIGA VFIGFLGAAG STMGAASVTL TVQARQLLSG IVQQQSNLLR AIEAQQHLLK LTVWGIKQLQ ARVLAVERYL KDQQLLGIWG
CSGKLICTTT VPWNSTWSNK SQEEIWGNMT WLQWDKEISN YTELIYSLIE ESQNQQEINE RDLLALDKWA NLWNWFDISN WLWYIKIFIV IVGGLIGLRI VFTVLSIINR VRQGYSPVSL QTLNPNPRDL DRPGRIEEEG GEQDRNRSIR LVSGFLALAW DDLRSLCLFS YHRLRDLILI AARTVELLGQ RGWEALKYLG NLAQYWVLEL KKSAISLFDT IAVAVAGWTD RVIEVVQGFG RALLHIPRRI RQGLERALL
R66M_07MAR06_F8
R66M_07MAR06_K7
R66M_07Mar06_N21
R66M_07Mar06_N22
R66M_07Mar06_O20
R66M_07Mar06_3A3
R66M_07Mar06_3A5
R66M_07Mar06_3A9
R66M_07Mar06_3B6
R66M_07Mar06_3C5
R66M_07Mar06_3D2
R66M_07Mar06_3D6
R66M_07Mar06_3G5
R66M_07Mar06_3H6
R66M_09AUG06_J8
R66M_09AUG06_K5
R66M_09AUG06_L4
R66M_09AUG06_L6
R66M_09AUG06_O5
